# Diet, cuisine and consumption practices of the first farmers in the southeastern Baltic

**DOI:** 10.1007/s12520-019-00804-9

**Published:** 2019-02-15

**Authors:** Harry K. Robson, Raminta Skipitytė, Giedrė Piličiauskienė, Alexandre Lucquin, Carl Heron, Oliver E. Craig, Gytis Piličiauskas

**Affiliations:** 1 BioArCh, Department of Archaeology, University of York, Heslington, York, YO10 5DD, UK; 2 Center for Physical Sciences and Technology, Saulėtekio Ave. 3, Vilnius 10257, Lithuania; 3 Lithuanian Institute of History, Kražių st. 5, Vilnius 01108, Lithuania; 4 Faculty of History, Vilnius University, Universiteto st. 7, Vilnius 01513, Lithuania; 5 Department of Scientific Research, The British Museum, Great Russell Street, London, WC1B 3DG, UK

**Keywords:** Southeastern Baltic, Neolithic, Bronze Age, Organic residue analysis, Aquatic biomarkers, Ceramic vessels

## Abstract

With the arrival of the Early Neolithic Globular Amphora and Corded Ware cultures into the southeastern Baltic, ca. 2900/2800–2400 cal BC, a new type of economy was introduced, animal husbandry. However, the degree to which this transformed the subsistence economy is unknown. Here, we conducted organic residue analyses of 64 ceramic vessels to identify their contents. The vessels were sampled from 10 Lithuanian archaeological sites dating across the Subneolithic-Neolithic transition to the Early Bronze Age (ca. 2900/2800–1300 cal BC). Our results demonstrate that regardless of location or vessel type, many ceramics were used to process aquatic resources. Against our expectations, this association continued even after marked economic change concurrent with the migration of pastoralists from central and southeastern Europe, as evidenced by recent ancient DNA analysis of human remains. Moreover, we observed dairy fats in pottery from all cultures of the Early Neolithic (i.e. Rzucewo, Globular Amphora and Corded Ware) but unlike other regions of Europe, it seems that these were incorporated into indigenous culinary practices. Furthermore, some vessels were used to process plant foods, and others may have been used for the production and/or storage of birch bark tar. However, evidence for domesticated plant processing, for example millet, was absent. We show that organic residue analysis of pottery provides a different picture of past consumption patterns compared to the stable isotope analysis of human remains from isolated burials where a clear dietary shift is evident.

**Electronic supplementary material:**

The online version of this article (10.1007/s12520-019-00804-9) contains supplementary material, which is available to authorized users.

## Introduction

The southeastern Baltic was one of the last regions in Europe to adopt agriculture,^1^ some 2500–1500 years after it was introduced in adjacent regions to the south. The reasons for this late adoption may seem obvious—the northerly latitudes were far less suitable for growing crops and rearing livestock, whilst indigenous wild game, fish and plants, which characterise the region, could easily have been procured from these densely forested, and highly productive aquatic ecotones (Piličiauskas 2018). It is perhaps harder to understand the eventual imposition of farming in this region, which is first recorded by the appearance of domesticated animals from ca. 2900/2800–2400 cal BC with the Early Neolithic Globular Amphora (GAC) and Corded Ware (CWC) cultures (Charniauski 1996; Lõugas et al. 2007; Piličiauskas et al. 2017a, b, c; Rimantienė 2002), and then by crop cultivation in the Middle Bronze Age, ca. 1300 cal BC (Piličiauskas et al. 2017c).

One obvious explanation for the arrival of farming in the southeastern Baltic is the northerly mass migration of pastoralists from regions to the south leading to the replacement of indigenous people and their economy. Indeed, analysis of ancient DNA (aDNA) from skeletal remains in the region demonstrates that both GAC and CWC populations shared some ancestry with central and southeastern Europeans. Whilst the GAC peoples from present day Poland and Ukraine are related to Anatolian farmers (Mathieson et al. 2018; Tassi et al. 2017), the CWC populations had an Eastern European steppe ancestry linking them to the pastoral communities of the Yamnaya Culture (Allentoft et al. 2015; Haak et al. 2015; Jones et al. 2017; Juras et al. 2018; Mittnik et al. 2018; Saag et al. 2017). Yet, the demise of such ‘affluent foragers’ of this region may not be simply explained by a population replacement by incoming farmers. The aDNA evidence also demonstrates that indigenous ‘forager’ populations resided and interacted with incoming ‘farmers’ until at least the mid-3rd millennium BC (Kristiansen et al. 2017; Mittnik et al. 2018), whilst the modern eastern Baltic population still possess the highest proportion of hunter-gatherer ancestry of all Europeans (Lazaridis et al. 2014; Malmström et al. 2009).

**Fig. 1:**
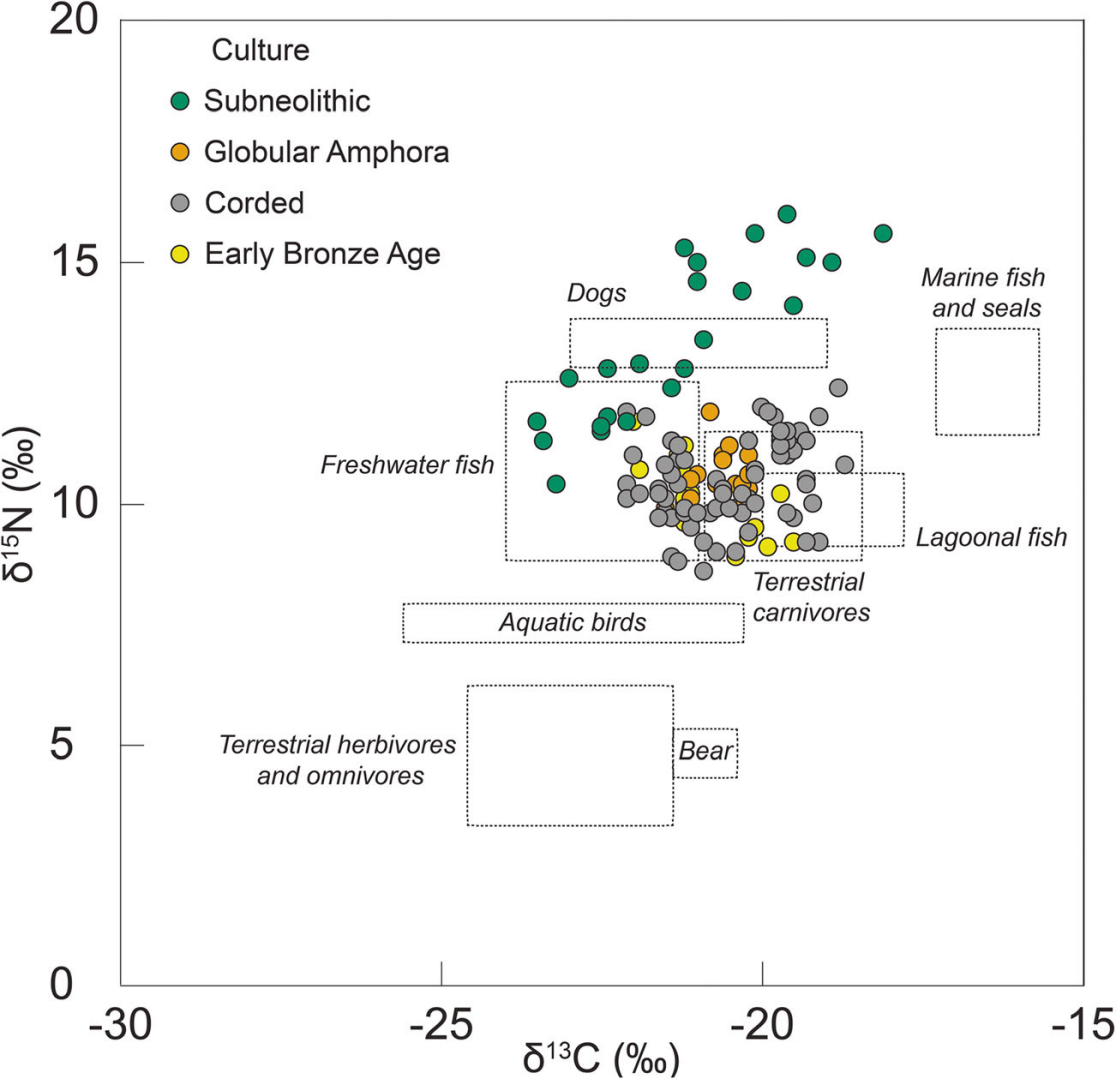
Carbon (δ^13^C) and nitrogen (δ^15^N) stable isotope data obtained on human (coloured circles) and faunal bone (dashed boxes) collagen from the southeastern Baltic and adjoining regions dating from the Subneolithic to the Early Bronze Age (data from Antanaitis and Ogrinc 2000; Antanaitis-Jacobs et al. 2009; Eriksson and Howcroft 2014; Eriksson et al. 2003, 2008; Fornander 2013; Heron et al. 2015; Laneman and Lang 2013; Piezonka et al. 2013; Piličiauskas et al. 2017a, b, 2018a; Reitsema 2012; Robson et al. 2016; Sjögren et al. 2016; Skipitytė unpublished data; Tõrv and Meadows 2015; this study) (Table S1)

Recently, stable isotope analysis of human bone collagen has been used to examine how the diets of incoming groups may have differed from indigenous populations. Data from the region (Fig. 1) shows a broad dietary change from aquatic-derived protein diets, with collagen enriched in ^15^N, in the Subneolithic to terrestrial-derived protein diets, depleted in ^15^N, with the appearance of the GAC and CWC (Antanaitis-Jacobs et al. 2009; Piličiauskas et al. 2017a, b, c). Nevertheless, it is difficult to quantify the degree of dietary change, and the approach does not completely exclude hunting and fishing during the GAC and CWC. The isotope dataset is also biased by the availability of human remains, which have largely been sampled from single burials or small cemeteries (Piličiauskas 2018) occupying distinct locations in the landscape, which may represent a possible funerary rite perhaps not afforded to all. For instance, despite the fact that there are over 70 CWC sites in Lithuania, there are only 20 known graves (Piličiauskas 2018). Moreover, supporting evidence from the analysis of botanical remains from settlement sites has been much less forthcoming due to the overall poor preservation of macroremains on ‘dry-land’ sites, and a lack of appropriate recovery techniques, especially for older excavations.

**Fig. 2:**
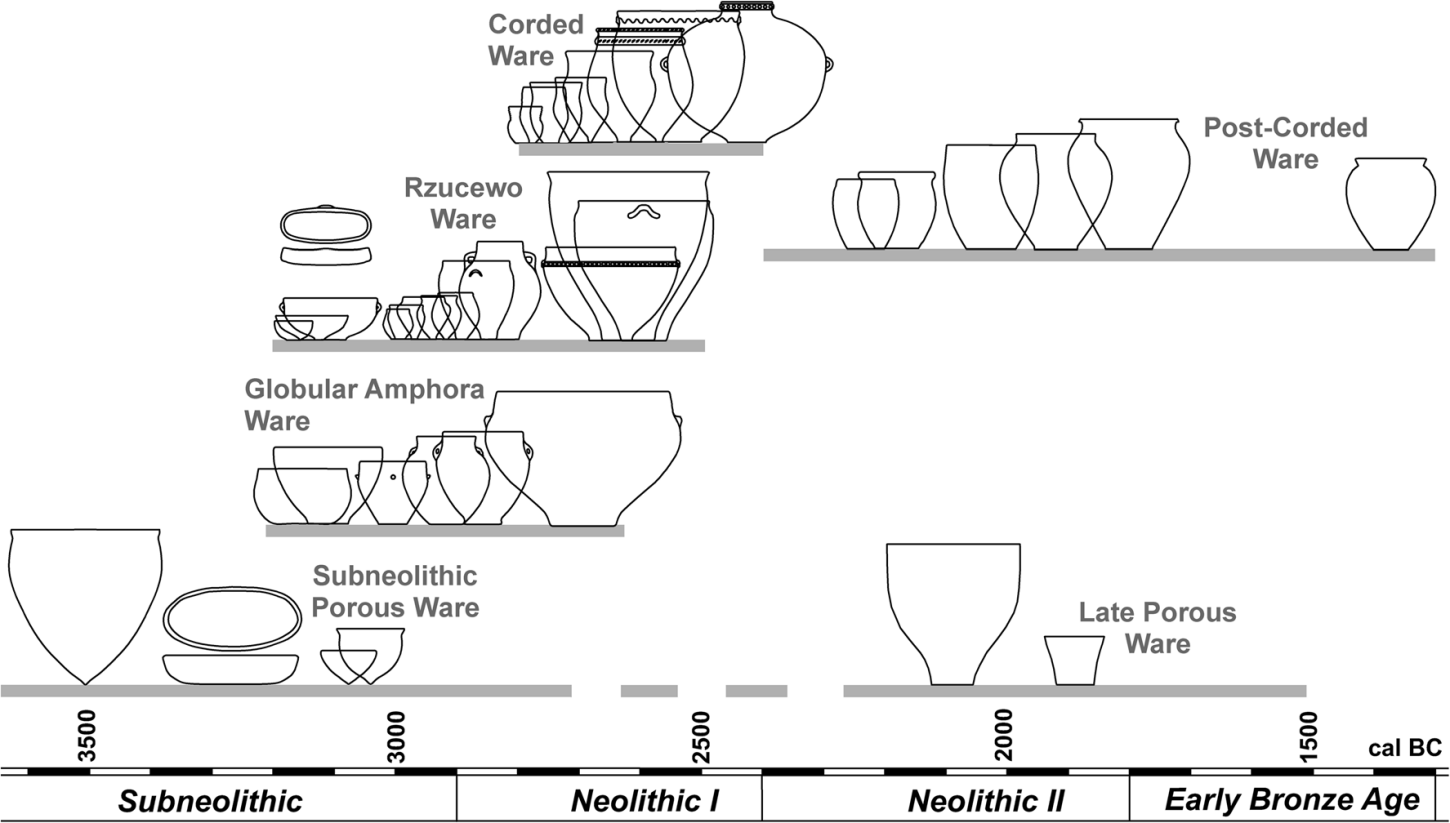
The pottery sequence in Lithuania. Adapted from Heron et al. (2015) and Piličiauskas (2016)

Organic residue analysis therefore offers an alternative approach for determining the types of foods commonly consumed and processed in the past, and this method is starting to be widely applied to pottery vessels throughout the Baltic region (e.g. Cramp et al. 2014a; Heron et al. 2015; Oras et al. 2017; Piličiauskas et al. 2018b). One advantage of this technique is based in part on the distinct ceramic typologies that have been confidently assigned to different cultural groups in the region (Fig. 2) (Piličiauskas 2016; Rimantienė 1984). In this region, Comb, Dubičiai, Narva, Neman and Porous wares were produced by Subneolithic hunter gatherers, whereas Rzucewo (RC), GAC and CWC wares are a characteristic of the Early Neolithic (Neolithic I) period. So-called Hybrid wares are defined as having a shell temper, which is a characteristic of Subneolithic pottery, but with ornamentation in the form of cord impressions that are usually associated with the Neolithic. This sequence is followed by more varied pottery types during the Late Neolithic (Neolithic II), and the Early Bronze Age (ca. 2400–1300 cal BC), which are commonly classified as post-Corded Wares. Another advantage is that large pottery assemblages are available from settlement sites throughout the region with many containing multiple typologies or stratified sequences offering the opportunity to examine cultural and temporal changes in utilitarian culinary practices.

## Materials and methods

### Sampled sites

**Fig. 3:**
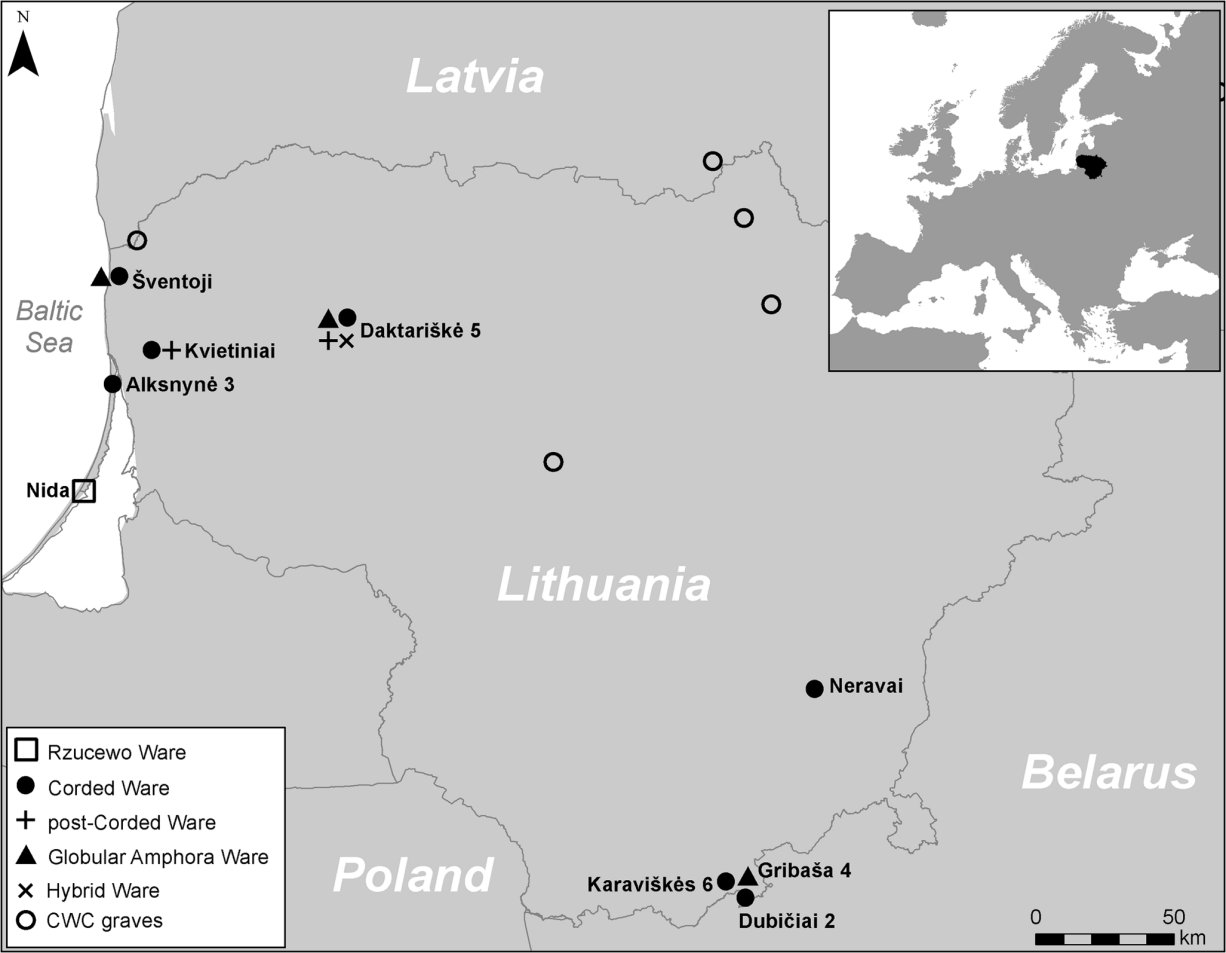
Map showing the locations of the sites sampled in this study and those sampled previously by Heron et al. (2015). In addition, all CWC burials previously sampled for stable isotope analysis are indicated (see “Discussion”). Insert, map showing the location of Lithuania in relation to Europe

In total, 10 archaeological habitation sites, with large pottery assemblages and thus representative vessels, situated in Lithuania were sampled (S1). In order to assess differences in pottery use, a range of sites situated in different ecotones were selected, i.e. coastal (estuarine/lagoonal), riverine and lacustrine (Fig. 3). The majority of the samples were selected from stratified or short-lived and well-dated sites (e.g. Alksnynė 3 and Daktariškė 5). In total, 28 vessels and 36 carbonised surface deposits, including interior ‘foodcrusts’ and exterior ‘sooted-crusts’, were sampled for organic residue analysis; the latter have recently been identified as food residues as opposed to soot from the fire used to heat the vessel contents (Piličiauskas et al. 2018b). A summary of the sampled vessels are provided in Table 1. The bulk carbon (δ^13^C) and nitrogen (δ^15^N) stable isotope data were complemented with data published elsewhere (Heron et al. 2015; Piličiauskas et al. 2018b).

**Table 1:** Summary of the samples analysed in this study

Location	Pottery typology (wares)	Period	Approximate radiocarbon dates (cal BC)	Number of vessels sampled
Alksnynė 3				
Lagoon	Corded	Neolithic I	2500/2400	10
Daktariškė 5				
Lakeshore	Globular Amphora	Neolithic I	2900–2700	5
Lakeshore	Corded	Neolithic I	2800–2400	10
Lakeshore	Post-Corded	Neolithic II—EBA	2400–1600	9
Lakeshore	Hybrid	Neolithic I?	2800–2400?	3
Dubičiai 2				
Lakeshore	Corded	Neolithic I	2800–2400	2
Gribaša 4				
Lakeshore	Globular Amphora	Neolithic I	2900–2400	2
Nida				
Lagoon	Rzucewo	Neolithic I	2900–2400	3
Karaviškės 6				
Lakeshore	Corded	Neolithic I	2800–2400	4
Kvietiniai				
River	Corded	Neolithic I	2800–2400	6
River	Post-Corded	Neolithic II—EBA	2000-1200	6
Neravai				
River	Corded	Neolithic I	2800–2400	1
Šventoji 1				
Lagoon	Corded	Neolithic I	2700–2400	1
Šventoji 4				
Lagoon	Globular Amphora	Neolithic I	2700	2
Total number of samples				64

### Bulk isotope ratio mass spectrometry analysis

Bulk δ^13^C and δ^15^N stable isotope analysis was undertaken on 36 carbonised surface deposits. Measurements were undertaken at two laboratories: the University of Bradford (UK) and the Centre for Physical Sciences and Technology, Vilnius (Lithuania). The samples were removed directly from the sherds using a scalpel, weighed out into tin capsules and then analysed without any pre-treatment. The samples were analysed in duplicate, and the values averaged where applicable. At Bradford, an ANCA-SL Elemental Analyser linked to a PDZ Europa 20/20 mass spectrometer was used. At Vilnius, an Elemental Analyser Flash EA1112 linked to a Thermo V Advantage Mass Spectrometer was employed. Calibrations and measurement uncertainty are discussed elsewhere (Piličiauskas et al. 2018b).

### Sampling for organic residue analysis

To reduce contamination from the burial environment, the surfaces of the potsherds were removed to a depth of between 1 and 2 mm using a Dremel drill fitted with a tungsten abrasive bit—this powder was then disposed of. Then, ca. 2 g of the ceramic powder was removed by drilling to a depth of between 2 and 4 mm from the interior surfaces of the potsherds. The powder was homogenised using a mortar and pestle. One aliquot was subjected to acidified methanol extraction followed by gas chromatography-mass spectrometry (GC-MS) and gas chromatography-combustion-isotope ratio mass spectrometry (GC-C-IRMS). A second aliquot was solvent extracted and analysed by GC-MS. When present, ca. 20 mg of the carbonised surface residues were removed from the vessel surface using a scalpel. These were homogenised using a mortar and pestle for acidified methanol extraction followed by GC-MS and GC-C-IRMS. A summary of the extraction procedure and analyses performed on each sample is provided in Table S2.

### Acidified methanol extraction

Lipids were directly extracted and methylated using an acidified methanol extraction procedure (Correa-Ascencio and Evershed 2014; Craig et al. 2013; Papakosta et al. 2015). Briefly, methanol (4 mL) was added to each sample. Then, the samples were ultrasonicated in a water bath for 15 min, acidified with concentrated sulphuric acid (800 μL), and vortexed and heated in closed vials for 4 h at 70 °C. After centrifugation, the acidified supernatants were transferred to sterilised and clean vials. Lipids were extracted with *n*-hexane (3 × 2 mL) and filtered through a pipette with potassium carbonate and glass wool to neutralise any sulphuric acid. The extracts were evaporated under a gentle stream of N_2_ at 37 °C. The samples were resuspended and transferred to a new vial, and then 10 μL of an internal standard (1.0 μg μL^−1^ hexatriacontane) was added to each sample before analysis by GC-MS and GC-C-IRMS using published methodologies (S2) (Craig et al. 2007, 2012; Hansel et al. 2004).

### Solvent extraction

Solvent extraction was undertaken using previously published methodologies (Craig et al. 2007; Dudd and Evershed 1998; Hansel and Evershed 2009). Briefly, 10 μL of an internal standard (*n*-tetratricontane) was added to each sample. Then, the samples were ultrasonicated for 15 min at 25 °C with three aliquots of dichloromethane:methanol (2:1 vol/vol; 5 mL). The extracts were combined and evaporated under a gentle stream of N_2_ at 37 °C to obtain a total lipid extract (TLE). Then, the samples were silylated with BSTFA + TMCS (99:1) at 70 °C for 1 h and evaporated to dryness under a gentle stream of N_2_. Derivatised samples were resuspended in *n*-hexane and directly analysed by GC-MS (S2).

### Instrumentation

The acidified methanol and solvent extracts were initially analysed by GC-MS (Agilent 7690A Series Gas Chromatograph coupled to an Agilent 5975C Inert XL Mass-Selective Detector with a Quadrupole Mass Analyser and Triple-Axis Detector (Agilent Technologies, Cheadle, Cheshire, UK)) using a DB-5-ms column ((5%-phenyl)-methylpolysiloxane column (30 m × 0.32 mm × 0.25 μm; J&W Scientific, Folsom, CA, USA)) in order to calculate the lipid yields and identify the main molecular compounds (S2). However, in order to explore the presence or absence of specific ions related to aquatic organisms, including 4,8,12-trimethyltridecanoic acid (TMTD), pristanic acid, phytanic acid and *ω*-(*o*-alkylphenyl) alkanoic acids (APAAs), the majority (49/64) of the acidified methanol extracts were analysed using a DB-23 ((50%-cyanopropyl)-methylpolysiloxane column (60 m × 0.25 mm × 0.25 μm; J&W Scientific, Folsom, CA, USA)) column in single-ion monitoring mode (SIM) (S2). In total, 18 solvent extracts were analysed by a HT-DB1 GC-MS-FID (100% dimethylpolysiloxane (15 m × 0.32 mm × 0.1 μm) (J&W Scientific, Folsom, CA, USA) column) to identify monoacylglycerols, triacylglycerols and diacylglycerols associated with plant products (S2), whilst 48 acidified methanol extracts were analysed by GC-C-IRMS (Delta V advantage isotope ratio mass spectrometer (Thermo Fisher Scientific, Bremen, Germany) linked to a Trace 1310 Gas Chromatograph (Thermo Fisher) with a ConFlo IV interface (CuO combustion reactor held at 850 °C)) to further distinguish the origins of the different foodstuffs (S2).

## Results

### Bulk carbon and nitrogen stable isotope analysis

**Fig. 4:**
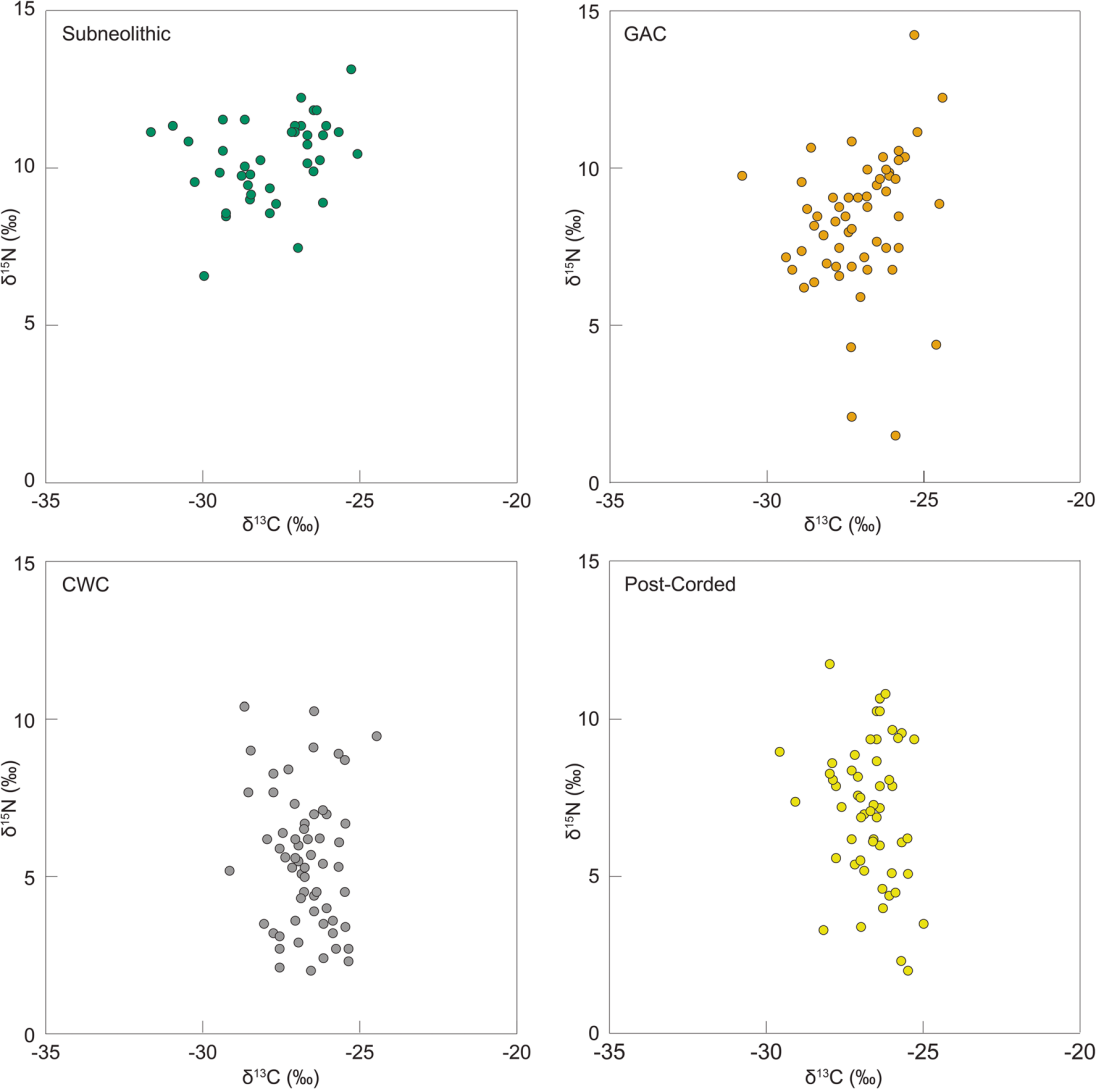
Bulk δ^13^C and δ^15^N stable isotope data obtained from carbonised surface residues (data from Heron et al. 2015; Piličiauskas et al. 2018b; this study). The data have been disaggregated according to ware (Table S3)

**Fig. 5:**
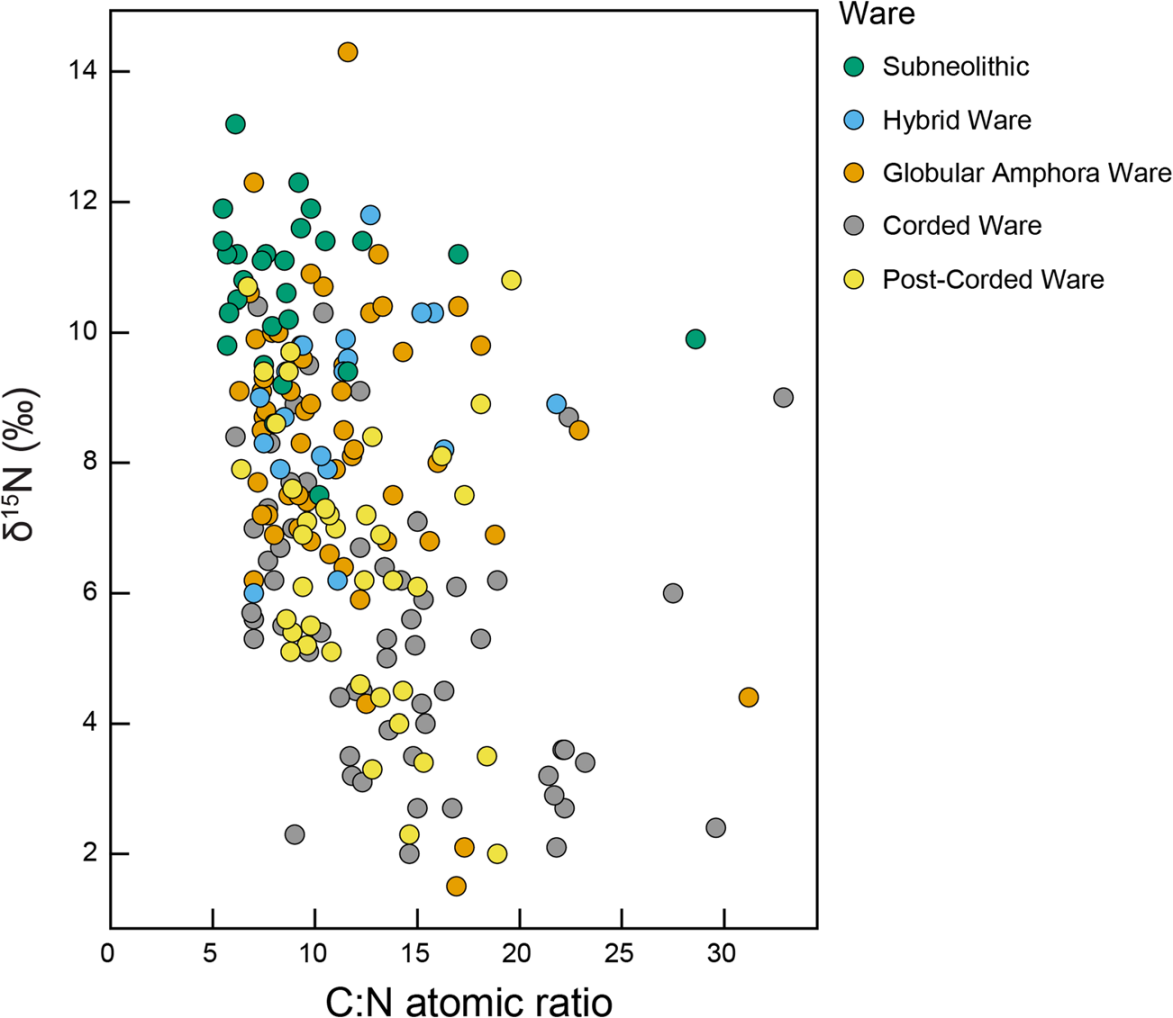
C:N atomic ratios and δ^15^N stable isotope data obtained from carbonised surface residues (data from Heron et al. 2015; Piličiauskas et al. 2018b; this study) (Table S3)

Bulk carbon (δ^13^C) and nitrogen (δ^15^N) stable isotope data were obtained on 36/64 vessels sampled in this study. These data were combined with data (*n* = 165) reported elsewhere (Heron et al. 2015; Piličiauskas et al. 2018b) (Table S3). Despite issues regarding the accuracy of this method (Heron and Craig 2015), δ^13^C values are typically higher in marine-derived residues compared to freshwater and terrestrial, whilst δ^15^N values are generally higher in aquatic-derived residues (> 6‰) compared to terrestrial (< 6‰; Craig et al. 2007). When the data were disaggregated according to period/ware (Fig. 4), there was a significant difference in the distribution of δ^15^N values between the groups (Kruskal-Wallis *χ*^2^ = 93.936; *p* = < 0.001). On the whole, the foodcrusts from the Subneolithic (i.e. Porous) and GAC vessels had higher δ^15^N values (1.5 to 14.4‰; mean = 9.1‰; *n* = 102) when compared with the CWC and post-Corded Ware vessels (2.0 to 10.8‰; mean = 5.9; *n* = 99) suggesting differences in their use. This was particularly evident for the CWC vessels derived from inland localities (2.0 to 10.4‰; mean = 5.0; *n* = 46) compared with coastal sites (3.6 to 10.3‰; mean = 7.2; *n* = 14), i.e. terrestrial resources in the hinterland compared with aquatic foodstuffs at the coast (Piličiauskas et al. 2018b). Interestingly, foodcrusts with lower δ^15^N values also tended to have higher quantities of carbon relative to nitrogen (i.e. high C:N atomic ratios; Pearson R = − 3.8; *p* = < 0.001) (Fig. 5). As plant products generally contain less protein, their contribution to the samples with higher C:N atomic ratios and lower δ^15^N values could be implied (Heron et al. 2016; Yoshida et al. 2013).

### Organic residue analysis

In total, 28/31 of the powdered ceramic sherds and 30/33 of the carbonised surface residues yielded sufficient quantities of lipids (Table S4), which were above the minimum amount required for interpretation (Evershed 2008; Craig et al. 2013).

### Results of the molecular analysis

The molecular results obtained by GC-MS are summarised in Table S4. In general, the residues are characterised by degraded animal fats with a high relative abundance of C_16:0_ and C_18:0_ unsaturated fatty acids and mono-, di- and triacylglycerols. A clear feature of the lipid profiles was that many of the samples (31/64) met the established criteria for the presence of aquatic biomarkers in archaeological pottery (Table S5), which includes APAAs with C_18_ and C_20_ carbon atoms, and at least one isoprenoid fatty acid, i.e. TMTD, pristanic or phytanic acid (Evershed et al. 2008a; Hansel et al. 2004). Since APAAs are only formed through extended heating of the pottery (Evershed et al. 2008a; Hansel et al. 2004), their presence confirms that they are derived from the use of the vessel. The ratio of the two naturally occurring phytanic acid diastereomers (%SSR) was also examined to further discriminate between aquatic and ruminant foodstuffs (Lucquin et al. 2016). Despite this, none of the samples could be securely assigned to either source as they yielded %SRR values that were within the range for both authentic aquatic oils and ruminant fats.

Molecular evidence for the processing of plant and insect products was infrequent even though a plant contribution to some vessels was supported by the bulk δ^13^C and δ^15^N stable isotope data (Fig. 5). In total, only a small proportion (13/64) of the sampled vessels yielded long-chain alkanes and traces of odd-chain fatty acids (Table S4), including C_29_, which may be derived from cuticles and the epicuticular waxes of plants (Raven et al. 1997) or alternatively beeswax (Regert et al. 2001). There was no discernible pattern regarding the presence of these compounds based on location, period/ware or vessel type, or indeed correspondence with the bulk stable isotope data, and some migration of these lipids from the soil cannot be ruled out given their low abundance in the potsherds. Triterpenoids, which are ubiquitously found in terrestrial and marine flora and fauna, were identified in 17/64 of the analysed samples (Table S4). These compounds could be derived from plant products that had been directly processed in the pottery or alternatively from sealing the vessels, wood smoke and even post-depositional contamination. Betulin and/or lupeol were present in a total of 13 vessels (Table S4) indicating the presence of birch bark (*Betula* sp.) resin (Pollard et al., 2016). Since both were identified in two exterior sooted crusts (DK87 I 4a and Dk 494), they may originate from the fuel used to heat the vessel contents rather than the production of birch bark tar for other purposes. In other cases, these compounds were present alongside animal fats indicating possible reuse of these vessels.

### Isotopic analysis of individual fatty acids

**Fig. 6:**
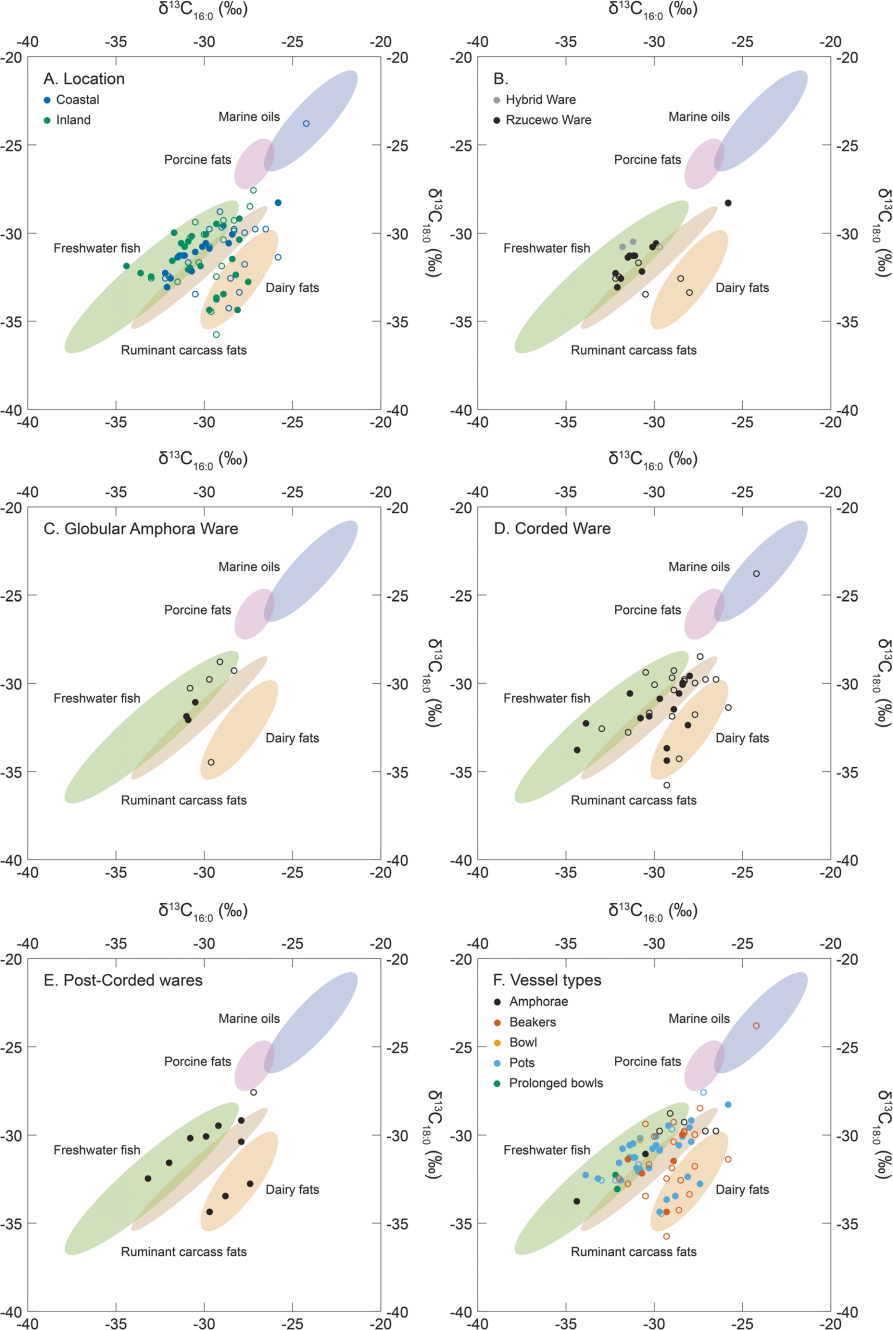
δ^13^C values of C_16:0_ and C_18:0_*n*-alkanoic acids extracted from 48/64 vessels analysed in this study alongside data (*n* = 27) obtained from contemporaneous vessels from the circum-Baltic region (data from Cramp et al. 2014a; Heron et al. 2015; Piličiauskas et al. 2018a). The ellipses are derived from modern authentic reference animals and are plotted at 68% confidence (Dudd 1999; Oras unpublished; Oras et al. unpublished; Pääkkönen et al. accepted). Closed circle—sample with aquatic biomarkers, open circle—sample without aquatic biomarkers

To distinguish vessel use further, 48/64 samples that yielded sufficient fatty acids were analysed by GC-C-IRMS. Carbon stable isotope (δ^13^C) values were obtained from the two fatty acid methyl esters, methyl palmitate (C_16:0_) and methyl stearate (C_18:0_) (Table S6). In Fig. 6, these data are plotted with data (Table S7) obtained from contemporaneous vessels (Cramp et al. 2014a; Heron et al. 2015; Piličiauskas et al. 2018a) as well as δ^13^C values obtained from modern authentic reference animal fats from the circum-Baltic region (Dudd 1999; Oras unpublished; Oras et al. unpublished; Pääkkönen et al. accepted).

In general, the vessels sampled from the coastal, including estuarine/lagoonal, sites yielded fatty acid δ^13^C values that generally support the presence of aquatic biomarkers, with the majority plotting within the range established for freshwater fish (Fig. 6a). There were some samples, however, that yielded aquatic biomarkers and plotted within the ranges established for ruminant carcass and dairy fats indicating that some mixing of vessel contents had taken place. Interestingly, many vessels sampled from the coastal sites were depleted in ^13^C when compared with data published from coastal sites elsewhere (Craig et al. 2007, 2011; Heron et al. 2013; Oras et al. 2017). This either indicates that freshwater fish from the Curonian Lagoon and Šventoji palaeolagoon or alternatively resources depleted in ^13^C from the Baltic Sea (Robson et al. 2016) had been processed in the vessels from the sites of Alksnynė 3, Nida and Šventoji 1 and 4. Organisms depleted in ^13^C could include seal (Phocidae), or fish that can tolerate a range of salinities, for example three-spined stickleback (*Gasterosteus aculeatus*).

When the data were disaggregated according to period/ware, some diachronic patterns emerge. Despite the range of fatty acid δ^13^C values (δ^13^C_16:0_ = − 32.2 to − 25.8‰, δ^13^C_18:0_ = − 33.5 to − 28.3‰) obtained from the RC vessels (Fig. 6b), only two had been solely used to process/store dairy fats; the remainder were used for processing freshwater fish and ruminant carcass fats and mixtures thereof, which was corroborated by the presence of aquatic biomarkers. Similarly, with one exception, the GAC wares were primarily used for processing freshwater fish and/or ruminant carcass fats (Fig. 6c). In contrast, the Corded (Fig. 6d) and post-Corded (Fig. 6e) wares had more varied use with vessels yielding fatty acid δ^13^C values indicative of freshwater fish, ruminant carcass and dairy fats. Overall, there was a significant difference (Kruskal-Wallis *χ*^2^ = 11.07; df = 4; *p* = 0.03) in Δ^13^C (δ^13^C_18:0_–δ^13^C_16:0_) between wares consistent with a greater frequency of terrestrial (ruminant carcass and dairy) derived fats in the Corded and post-Corded Ware vessels when compared with the other Early Neolithic ceramics (i.e. RC and GAC). There was only one CWC vessel that plotted within the range for marine oils (Fig. 6d), a beaker from the Finnish site of Kirkkonummi Koivistosveden (Cramp et al. 2014a), and one post-Corded Ware pot that may have been used to process porcine fats (Fig. 6e), a pot from the Lithuanian site of Kvietiniai. The one bowl and two prolonged bowls, the latter which have been interpreted as lamps (Heron et al. 2015; Oras et al. 2017), indicated the processing of freshwater fish. Lastly, although many of the pots yielded aquatic biomarkers they plotted within the freshwater fish, ruminant carcass and dairy fat ranges demonstrating that some mixing of vessel contents had taken place.

## Discussion

### Continuity in pottery use with the appearance of domesticates

Our analyses provide new insights into changing culinary practices with the arrival of domesticated animals with the Early Neolithic CWC and GAC. Previous studies have shown that aquatic resources were extensively processed in hunter-gatherer ceramics throughout the region (Heron et al. 2015; Oras et al. 2017; Piličiauskas et al. 2018b). The isotope and molecular evidence combined show that the Early Neolithic ceramics from all cultures (i.e. RC, GAC and CWC) were used for the processing of aquatic resources, regardless of location or vessel type, compelling evidence for continuity in pottery use beyond the Neolithic transition. Except for the RC wares (Fig. 6b), the youngest samples, dating to the Late Neolithic (Neolithic II) and Early Bronze Age, i.e. post-Corded Wares, had the highest frequency of aquatic biomarkers (Fig. 6e) compared to the other Early Neolithic ceramics. In the majority of cases, the isotope data suggest these were organisms from either freshwater or brackish environments that characterise the region. Here, there is no evidence that pottery use radically changed with the introduction of domesticated animals.

Intriguingly, the human stable isotope record shows dietary shift in the Neolithic period away from aquatic resources. One explanation for this discrepancy is that pottery served a wider community than those represented by the burials. Burials may represent a small proportion of society, marking a funerary rite that may not have been afforded by all, and their discovery and excavation is often limited to specific localities. For example, all CWC individuals from Lithuania that have been analysed using stable isotopes (i.e. Benaičiai, Biržai, Plinkaigalis, Gyvakarai; Piličiauskas et al. 2017a, 2018a) are from graves situated in river valleys whereas pottery producing habitation sites are usually located on coastlines and lakeshores (Fig. 3). Finally, it should be noted that the stable isotope analysis of Neolithic individuals does not rule out some consumption of aquatic-derived foods and may be interpreted as dietary broadening to encompass terrestrial products, which is evident through pottery use, especially the CWC ceramics. Equally, food processed in pottery vessels do not necessarily accurately reflect diet.

### The appearance of dairying with the Corded Ware culture

Dairy fats are frequently identified in prehistoric pottery throughout Europe, and this practice is likely to have formed an important part of the economy as soon as domesticated animals were introduced (Copley et al. 2003; Craig et al. 2005; Debono Spiteri et al. 2016; Dunne et al. 2012; Evershed et al. 2008b). Whilst the southeastern Baltic seems no exception, the frequency of ruminant carcass and dairy fats is much lower than reported in other studies of the Early Neolithic ceramic use due to the fact that aquatic resources continued to be processed. Interestingly, it has been shown through aDNA analysis that selection for the gene conferring the ability of adults to digest the sugars in fresh milk (i.e. lactose persistence) arose during the formation of the CWC (Allentoft et al. 2015) corresponding to the introduction of domesticated animals to this region. Previously, it has been suggested that dairy products are associated with Corded Ware beaker-type ‘drinking’ vessels over other vessel forms (Cramp et al. 2014a; Heron et al. 2015; Piličiauskas et al. 2018a), which would support this example of gene-culture evolution.

**Fig. 7:**
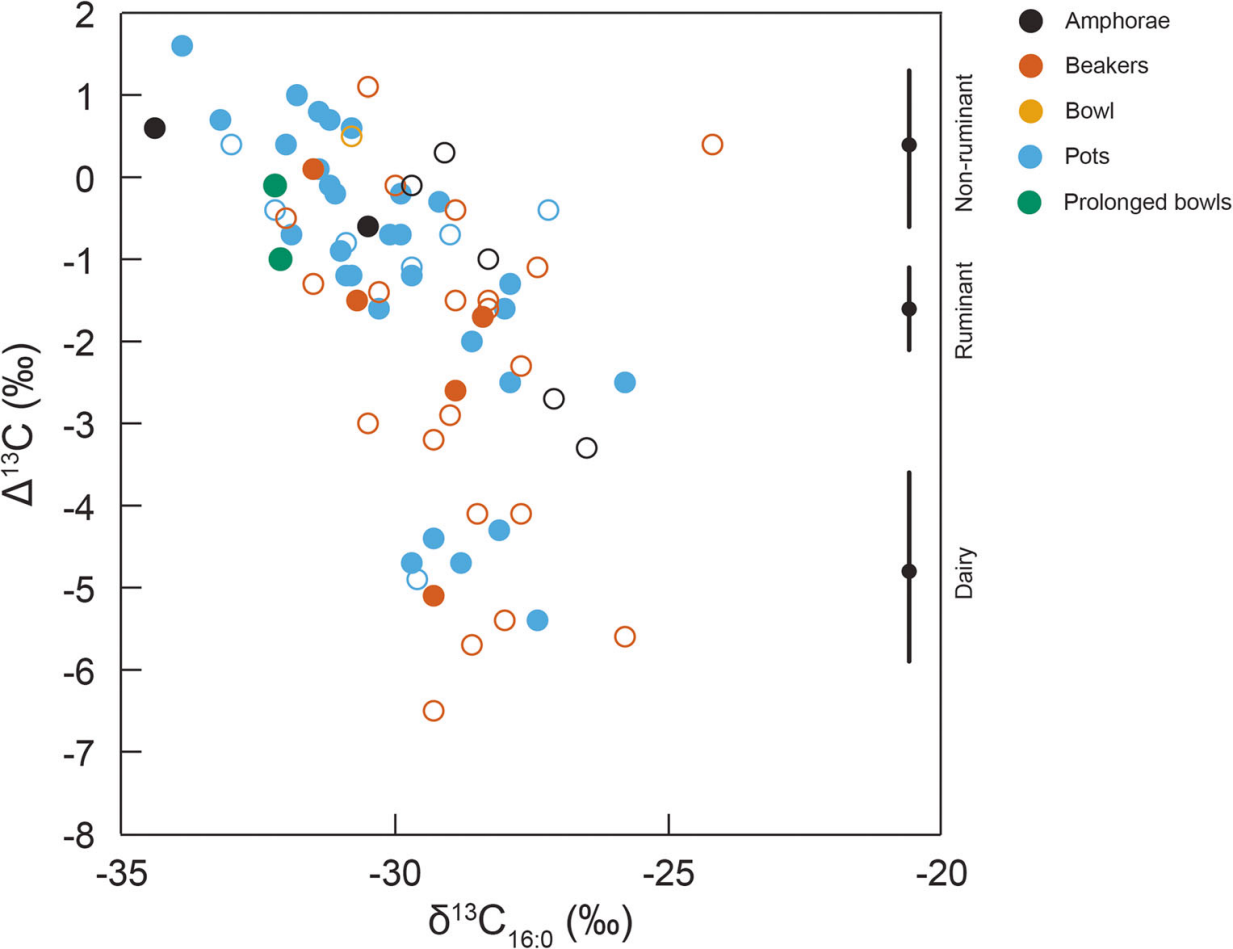
Difference in the δ^13^C isotope values (Δ^13^C) between individual C_18:0_ and C_16:0_*n*-alkanoic acids extracted from 48/64 vessels analysed in this study alongside data (*n* = 27) obtained from contemporaneous vessels from the circum-Baltic region (data from Cramp et al. 2014a; Heron et al. 2015; Piličiauskas et al. 2018a). The reference ranges, including the median, are derived from modern authentic reference animals (Dudd 1999; Oras unpublished; Oras et al. unpublished; Pääkkönen et al. accepted). Closed circle—sample with aquatic biomarkers, open circle—sample without aquatic biomarkers

However, when the Lithuanian data are considered, there is no preference for the processing of dairy fats in Corded Ware vessels compared to other Early Neolithic cultures (*χ*^2^ test of independence (group vs presence/absence of dairy) = 2.9981; df = 4; *p* value = 0.559) nor is there any association with dairy fats and vessel type (Fig. 7). Whether dairy products were the preserve of migrants with ancestry in the Yamnaya culture is therefore debatable. Fresh milk can be easily transformed into low-lactose dairy products, such as cheese and yoghurt, and therefore available to all, which may explain their presence more broadly in European Early Neolithic pottery. The analysis of Lithuanian Neolithic and Early Bronze Age ceramic vessels also suggests that dairy fats were mixed with other products, including aquatic foodstuffs (Fig. 6e). Rather than afforded special treatment, it seems likely that dairy products were incorporated into a broader cuisine that in part is derived from previous hunter-gatherer practices.

### Comparison with the zooarchaeological evidence

Although it is often fruitful to compare the data obtained from organic residue analysis with the zooarchaeological evidence (e.g. Oras et al. 2017; Pääkkönen et al. 2016), faunal remains are not well preserved at the majority of Lithuanian Neolithic and Early Bronze Age sites due to the acidity of the soils. When animal bones are present, they are often burnt and highly fragmented (< 1 cm), as in the case of Dubičiai 2, Gribaša 4, Karaviškės 6, Kvietiniai and Neravai, or difficult to assign to a single cultural phase, as in the case of Daktariškė 5, Šarnelė, Šventoji 1, 2, 4 and 6. In cases where zooarchaeological remains are better preserved and were found at relatively short-term sites (e.g. Alksnynė 3, Kretuonas 1C and Nida), they indicate a mixed economy, which incorporated fishing, hunting and animal husbandry (Daugnora and Girininkas 2004; Piličiauskas 2018; Piličiauskas et al. 2017c, 2018a). For instance, at the RC site of Nida, the zooarchaeological evidence demonstrates a mixed economy with a focus on fishing. Here, a range of domesticated and wild mammalian taxa was recovered, which were found alongside freshwater, anadromous and marine fish (Piličiauskas 2018; Piličiauskienė unpublished data; Schmölcke unpublished data). Similarly, at the CWC site of Alksnynė 3 located nearby, the faunal assemblage demonstrated animal husbandry, freshwater fishing from the Curonian Lagoon and seal hunting (Piličiauskas 2018). Moreover, at the post-Corded Porous Ware site of Kretuonas 1C (ca. 1900–1600 cal BC), located in the hinterland, an assemblage composed of domesticated and wild mammalian taxa was recovered alongside numerous fish remains dominated by northern pike (*Esox lucius*) (NISP = 529) (Daugnora and Girininkas 2004). Whilst the aforementioned examples demonstrate a mixed economy for the Late Porous Ware (or Late Narva), RC and CWC cultures, it is not possible to characterise the subsistence economy of the GAC with certainty given the generally poor stratigraphic control at many sites (e.g. Daktariškė 5, Šventoji 2, 4 and 6). Regardless, our data corroborate the zooarchaeological evidence demonstrating the processing of domestic and wild animals and their resources in Early Neolithic pottery.

## Conclusions

The new data presented here adds to a growing corpus of data concerning pottery use throughout Europe during the Early Neolithic where some broad trends can be outlined. Where farming and new forms of pottery are introduced to regions occupied by ceramic hunter gatherers, a hybridisation of pottery use emerges that incorporates both wild aquatic foods and domesticated resources, for instance across the Ertebølle and Funnel Beaker transition in the western Baltic (Craig et al. 2007, 2011). In contrast, the majority of pottery used by Neolithic farmers in regions previously occupied by aceramic hunter gatherers was solely used for domestic terrestrial resources, including those from coastal sites, for example the UK (Cramp et al. 2014b) and Mediterranean (Debono Spiteri et al. 2016). One reason for this is that ceramic hunter gatherers generally occupied rich aquatic ecotones, which may be associated with a long-standing tradition of pottery use, for instance the Narva culture (Kriiska et al. 2017; Oras et al. 2017).

Organic residue analysis of over 60 ceramic vessels from throughout Lithuania demonstrates that many of the samples (31/64) were primarily used to process aquatic resources regardless of location (coastal or inland) or vessel type (amphorae, beakers, pots). Interestingly, many of the youngest samples in the dataset, i.e. post-Corded Wares, yielded aquatic biomarkers. Based on these data, we propose that the association between pottery use and aquatic resources may have been intrinsically linked culturally as has been demonstrated elsewhere (Gibbs et al. 2017; Lucquin et al. 2018; Tache and Craig 2015). Moreover, despite demographic change as demonstrated by aDNA analysis (Allentoft et al. 2015; Haak et al. 2015; Jones et al. 2017; Juras et al. 2018; Mittnik et al. 2018; Saag et al. 2017), the introduction of domesticated plants and animals appears not to have transformed economies equally throughout Europe. Initially, it seems likely that incoming CWC pastoralists resided side-by-side with indigenous hunter gatherers before elements of the Neolithic package, for instance dairy products, were integrated into their lifeway’s. However, analyses of further CWC vessels are required to determine whether this practice was pervasive throughout the occupied region.

## Electronic supplementary material

ESM 1 (DOCX 246 kb)

ESM 2 (DOCX 38.2 kb)

## Footnotes

1 In this contribution, agriculture refers to the practice of farming, which includes land cultivation for the growing of crops and the rearing of livestock. These terms primarily refer to the GAC and CWC who are culturally linked to the central European Neolithic.
