# Supplementary material for: Diet, cuisine and consumption practices of the first farmers in the southeastern Baltic
Source: Archaeol Anthropol Sci. 2019 Feb 15;11(8):4011–24. doi: 10.1007/s12520-019-00804-9 (PMC6743674; doi:10.1007/s12520-019-00804-9)
Supplement: Supplementary file 1 — (DOCX 246 kb) [file 12520_2019_804_MOESM1_ESM.docx]

**Supplementary information for Diet, cuisine and consumption practices of the first farmers in the south-eastern Baltic**

Harry K. Robson^1*^, Raminta Skipitytė^2, 5^, Giedrė Piličiauskienė^3^, Alexandre Lucquin^1^, Carl Heron^4^, Oliver E. Craig^1^ and Gytis Piličiauskas^5*^

¹BioArCh, Department of Archaeology, University of York, Heslington, York YO10 5DD, UK

^2^Center for Physical Sciences and Technology, Saulėtekio ave. 3, Vilnius 10257, Lithuania

^3^Faculty of History, Vilnius University, Universiteto st. 7, Vilnius 01513, Lithuania

^4^Department of Scientific Research, The British Museum, Great Russell Street, London WC1B 3DG, UK

^5^Lithuanian Institute of History, Kražių st. 5, Vilnius 01108, Lithuania

*corresponding authors: hkrobson@hotmail.co.uk and gytis.piliciauskas@gmail.com

**This word document includes:**

Tables S1 to S7

***Table S1. Carbon (δ^13^C) and nitrogen (δ^15^N) stable isotope data obtained on human and faunal bone collagen. Blank – data not reported.***

| **Country** | **Location** | **Site** | **Period** | **Species** | **δ^13^C** | **δ^15^N** | **C:N atomic**  **ratio** | **Reference** |
| --- | --- | --- | --- | --- | --- | --- | --- | --- |
| Lithuania | Inland | Spiginas | Subneolithic | Human | -22.4 | 12.8 | 3.3 | Piličiauskas et al. 2017c |
| Lithuania | Inland | Donkalnis | Subneolithic | Human | -23.5 | 11.7 | 3.3 | Piličiauskas et al. 2017c |
| Lithuania | Inland | Donkalnis | Subneolithic | Human | -22.5 | 11.5 | 3.3 | Piličiauskas et al. 2017c |
| Lithuania | Coastal | Šventoji 2/4 | Subneolithic/Neolithic | Human | -20.1 | 15.6 | 3.2 | Piličiauskas et al. 2017b |
| Lithuania | Coastal | Šventoji 23 | Subneolithic | Human | -18.9 | 15.0 | 3.3 | Piličiauskas et al. 2017b |
| Lithuania | Coastal | Šventoji 23 | Subneolithic | Human | -19.3 | 15.1 | 3.3 | Piličiauskas et al. 2017b |
| Lithuania | Coastal | Šventoji 23 | Subneolithic | Human | -20.3 | 14.4 | 3.4 | Piličiauskas et al. 2017b |
| Lithuania | Coastal | Šventoji 23 | Subneolithic | Human | -19.6 | 16.0 | 3.3 | Piličiauskas et al. 2017b |
| Lithuania | Coastal | Šventoji 23 | Subneolithic | Human | -21.0 | 15.0 | 3.3 | Piličiauskas et al. 2017b |
| Lithuania | Coastal | Šventoji 23 | Subneolithic | Human | -21.2 | 15.3 | 3.2 | Piličiauskas et al. 2017b |
| Lithuania | Inland | Daktariškė 1 | Subneolithic | Human | -21.9 | 12.9 | 3.3 | Piličiauskas et al. 2017c |
| Lithuania | Coastal | Šventoji 43 | Subneolithic | Horse | -24.7 | 5.7 | 3.5 | Piličiauskas et al. 2017b |
| Lithuania | Inland | Kretuonas 1B | Subneolithic | Human | -23.4 | 11.3 | 3.2 | Antanaitis and Ogrinc 2000 |
| Lithuania | Inland | Kretuonas 1B | Subneolithic | Human | -22.4 | 11.8 | 3.2 | Piličiauskas et al. 2017c |
| Lithuania | Coastal | Šventoji 43 | Subneolithic | Auroch/bison | -22.4 | 3.4 | 3.3 | Piličiauskas et al. 2017b |
| Lithuania | Coastal | Šventoji 2/4 | Subneolithic | Auroch/bison | -22.6 | 5.1 | 3.1 | Piličiauskas et al. 2017b |
| Lithuania | Coastal | Šventoji 43 | Subneolithic | Horse | -23.2 | 4.9 | 3.2 | Piličiauskas et al. 2017b |
| Lithuania | Coastal | Šventoji 43 | Subneolithic | Elk | -23.5 | 4.4 | 3.3 | Piličiauskas et al. 2017b |
| Lithuania | Coastal | Šventoji 43 | Subneolithic | Bear | -21.4 | 5.7 | 3.3 | Piličiauskas et al. 2017b |
| Lithuania | Coastal | Šventoji 43 | Subneolithic | Seal | -16.5 | 12.7 | 3.3 | Piličiauskas et al. 2017b |
| Lithuania | Coastal | Šventoji 3 | Subneolithic | Seal | -15.6 | 11.3 | 3.2 | Piličiauskas et al. 2017b |
| Lithuania | Coastal | Šventoji 43 | Subneolithic | Seal | -16.9 | 11.7 | 3.4 | Piličiauskas et al. 2017b |
| Lithuania | Coastal | Šventoji 43 | Subneolithic | Roe deer | -23.5 | 4.5 | 3.2 | Piličiauskas et al. 2017b |
| Lithuania | Coastal | Šventoji 43 | Subneolithic | Dog | -23.8 | 13.9 | 3.5 | Piličiauskas et al. 2017b |
| Lithuania | Coastal | Šventoji 43 | Subneolithic | Boar/pig | -22.2 | 6.9 | 3.2 | Piličiauskas et al. 2017b |
| Lithuania | Inland | Žemaitiškė 2 | Subneolithic | Cattle | -22.7 | 4.8 | 3.2 | Piličiauskas et al. 2017b |
| Lithuania | Inland | Žemaitiškė 2 | Subneolithic | Bear | -20.9 | 4.6 | 3.2 | Piličiauskas et al. 2017c |
| Lithuania | Inland | Žemaitiškė 2 | Subneolithic | Bear | -20.1 | 4.7 | 3.2 | Piličiauskas et al. 2017c |
| Lithuania | Inland | Žemaitiškė 2 | Subneolithic | Red deer | -23.2 | 4.2 | 3.2 | Piličiauskas et al. 2017c |
| Lithuania | Inland | Žemaitiškė 2 | Subneolithic | Red deer | -22.3 | 5.4 | 3.2 | Piličiauskas et al. 2017c |
| Lithuania | Inland | Žemaitiškė 2 | Subneolithic | Beaver | -22.0 | 5.5 | 3.3 | Piličiauskas et al. 2017c |
| Lithuania | Inland | Žemaitiškė 2 | Subneolithic | Beaver | -22.6 | 5.6 | 3.3 | Piličiauskas et al. 2017c |
| Lithuania | Inland | Žemaitiškė 2 | Subneolithic | Auroch/bison | -22.6 | 5.1 | 3.3 | Piličiauskas et al. 2017c |
| Lithuania | Inland | Žemaitiškė 2 | Subneolithic | Auroch/bison | -22.9 | 5.4 | 3.3 | Piličiauskas et al. 2017c |
| Lithuania | Inland | Žemaitiškė 2 | Subneolithic | Boar | -23.1 | 4.6 | 3.2 | Piličiauskas et al. 2017c |
| Lithuania | Inland | Žemaitiškė 2 | Subneolithic | Boar | -22.4 | 4.1 | 3.2 | Piličiauskas et al. 2017c |
| Lithuania | Inland | Žemaitiškė 2 | Subneolithic | Elk | -23.1 | 4.1 | 3.3 | Piličiauskas et al. 2017c |
| Lithuania | Inland | Žemaitiškė 2 | Subneolithic | Elk | -22.9 | 4.1 | 3.3 | Piličiauskas et al. 2017c |
| Lithuania | Inland | Žemaitiškė 2 | Subneolithic | Boar/pig | -21.4 | 3.8 | 3.3 | Piličiauskas et al. 2017c |
| Lithuania | Coastal | Šventoji 52 | Subneolithic | Wolf | -20.9 | 9.0 | 3.2 | Piličiauskas et al. 2017b |
| Lithuania | Coastal | Šventoji 2/4 | Subneolithic | Pike | -22.1 | 9.5 | 3.3 | Piličiauskas et al. 2017b |
| Lithuania | Coastal | Šventoji 2/4 | Subneolithic | Pikeperch | -22.6 | 10.9 | 3.4 | Piličiauskas et al. 2017b |
| Lithuania | Coastal | Šventoji 2/4 | Subneolithic | Perch | -20.0 | 10.6 | 3.3 | Piličiauskas et al. 2017b |
| Lithuania | Coastal | Šventoji 23 | Subneolithic | Human | -18.1 | 15.6 | 3.2 | Piličiauskas et al. 2017b |
| Lithuania | Inland | Donkalnis | Late Mesolithic/Subneolithic | Human | -23.2 | 10.4 | 3.3 | Antanaitis-Jacobs et al. 2009 |
| Lithuania | Inland | Donkalnis | Subneolithic | Human | -22.1 | 11.7 | 3.3 | Antanaitis-Jacobs et al. 2009 |
| Lithuania | Coastal | Šventoji 2/4 | Subneolithic | Elk | -23.6 | 4.9 | 3.3 | Antanaitis-Jacobs et al. 2009 |
| Lithuania | Coastal | Šventoji 1B | Subneolithic | Elk | -23.1 | 3.6 | 3.4 | Antanaitis-Jacobs et al. 2009 |
| Lithuania | Inland | Žemaitiškė 1 | Subneolithic | Red deer | -24.1 | 4.0 | 3.5 | Antanaitis-Jacobs et al. 2009 |
| Lithuania | Inland | Žemaitiškė 3B | Subneolithic | Bear | -20.7 | 5.0 | 3.3 | Antanaitis-Jacobs et al. 2009 |
| Lithuania | Coastal | Šventoji 3 | Subneolithic | Bear | -21.4 | 4.5 | 3.3 | Antanaitis-Jacobs et al. 2009 |
| Lithuania | Coastal | Šventoji 6 | Subneolithic | Bear | -20.9 | 4.4 | 3.5 | Antanaitis-Jacobs et al. 2009 |
| Lithuania | Inland | Kretuonas 1B | Subneolithic | Boar/pig | -23.6 | 6.4 | 3.5 | Antanaitis-Jacobs et al. 2009 |
| Lithuania | Coastal | Šventoji 1B | Subneolithic | Boar/pig | -21.8 | 4.1 | 3.4 | Antanaitis-Jacobs et al. 2009 |
| Lithuania | Coastal | Šventoji 3 | Subneolithic | Boar/pig | -21.3 | 3.8 | 3.3 | Antanaitis-Jacobs et al. 2009 |
| Lithuania | Coastal | Šventoji 3 | Subneolithic | Boar | -21.7 | 5.5 | 3.4 | Antanaitis-Jacobs et al. 2009 |
| Lithuania | Coastal | Šventoji 6 | Subneolithic | Boar | -21.6 | 5.3 | 3.4 | Antanaitis-Jacobs et al. 2009 |
| Lithuania | Inland | Žemaitiškė 1 | Subneolithic | Boar | -23.3 | 6.5 | 3.5 | Antanaitis-Jacobs et al. 2009 |
| Lithuania | Coastal | Šventoji 23 | Subneolithic | Mallard | -21.1 | 7.8 | 3.5 | Antanaitis-Jacobs et al. 2009 |
| Lithuania | Inland | Žemaitiškė 2 | Subneolithic | Common goldeneye | -23.3 | 7.3 | 3.4 | Antanaitis-Jacobs et al. 2009 |
| Lithuania | Coastal | Šventoji 23 | Subneolithic | Wood grouse | -21.9 | 2.2 | 3.6 | Antanaitis-Jacobs et al. 2009 |
| Lithuania | Coastal | Šventoji 2/4 | Subneolithic | Pike | -21.6 | 12.6 | 3.3 | Antanaitis-Jacobs et al. 2009 |
| Lithuania | Coastal | Šventoji 2/4 | Subneolithic | Pikeperch | -21.8 | 12.6 | 3.5 | Antanaitis-Jacobs et al. 2009 |
| Lithuania | Coastal | Šventoji 2/4 | Subneolithic | Flounder | -16.6 | 11.6 | 3.3 | Antanaitis-Jacobs et al. 2009 |
| Lithuania | Coastal | Šventoji 3 | Subneolithic | Beaver | -22.1 | 5.4 | 3.4 | Antanaitis-Jacobs et al. 2009 |
| Lithuania | Inland | Žemaitiškė 2 | Subneolithic | Beaver | -23.9 | 4.8 | 3.3 | Antanaitis-Jacobs et al. 2009 |
| Lithuania | Inland | Žemaitiškė 2 | Subneolithic | Beaver | -23.0 | 3.5 | 3.5 | Antanaitis-Jacobs et al. 2009 |
| Lithuania | Coastal | Šventoji 23 | Subneolithic | Otter (seal?) | -16.5 | 13.8 | 3.4 | Antanaitis-Jacobs et al. 2009 |
| Lithuania | Coastal | Šventoji 2/4 | Subneolithic | Seal | -17.7 | 10.6 | 3.4 | Antanaitis-Jacobs et al. 2009 |
| Lithuania | Coastal | Šventoji 2/4 | Subneolithic | Seal | -16.3 | 12.2 | 3.4 | Antanaitis-Jacobs et al. 2009 |
| Lithuania | Coastal | Šventoji 2/4 | Subneolithic | Seal | -16.1 | 12.0 | 3.4 | Antanaitis-Jacobs et al. 2009 |
| Lithuania | Coastal | Šventoji 1B | Subneolithic | Seal | -15.5 | 13.1 | 3.3 | Antanaitis-Jacobs et al. 2009 |
| Lithuania | Coastal | Šventoji 2/4 | Subneolithic | Seal | -18.7 | 13.9 | 3.4 | Antanaitis-Jacobs et al. 2009 |
| Lithuania | Coastal | Šventoji 2/4 | Subneolithic | Seal | -15.8 | 12.4 | 3.3 | Antanaitis-Jacobs et al. 2009 |
| Lithuania | Coastal | Šventoji 1B | Subneolithic | Seal | -16.5 | 11.1 | 3.4 | Antanaitis-Jacobs et al. 2009 |
| Lithuania | Coastal | Šventoji 6 | Subneolithic | Seal | -17.1 | 12.6 | 3.4 | Antanaitis-Jacobs et al. 2009 |
| Lithuania | Coastal | Šventoji 6 | Subneolithic | Seal | -16.6 | 13.3 | 3.4 | Antanaitis-Jacobs et al. 2009 |
| Lithuania | Coastal | Šventoji 23 | Subneolithic | Seal | -16.5 | 12.7 | 3.5 | Antanaitis-Jacobs et al. 2009 |
| Lithuania | Coastal | Šventoji 6 | Subneolithic | Dog | -20.7 | 13.3 | 3.4 | Antanaitis-Jacobs et al. 2009 |
| Lithuania | Coastal | Šventoji 23 | Subneolithic | Dog | -19.2 | 12.8 | 3.6 | Antanaitis-Jacobs et al. 2009 |
| Lithuania | Coastal | Šventoji 3 | Subneolithic | Fox | -18.5 | 11.4 | 3.4 | Antanaitis-Jacobs et al. 2009 |
| Lithuania | Inland | Žemaitiškė 2 | Subneolithic | Marten | -20.1 | 8.8 | 3.5 | Antanaitis-Jacobs et al. 2009 |
| Lithuania | Inland | Žemaitiškė 2 | Subneolithic | Badger | -19.5 | 10.1 | 3.6 | Antanaitis-Jacobs et al. 2009 |
| Lithuania | Coastal | Šventoji 2/4 | Subneolithic | Mallard | -24.8 | 7.2 | 3.3 | Antanaitis-Jacobs et al. 2009 |
| Lithuania | Coastal | Šventoji 2/4 | Subneolithic | Seal | -16.3 | 15.5 | 3.4 | Heron et al. 2015 |
| Lithuania | Coastal | Šventoji 2/4 | Subneolithic | Seal | -15.3 | 13.1 | 3.2 | Heron et al. 2015 |
| Lithuania | Coastal | Šventoji 2/4 | Subneolithic | Seal | -16.6 | 12.0 | 3.2 | Heron et al. 2015 |
| Lithuania | Coastal | Šventoji 6 | Subneolithic | Human | -19.5 | 14.1 | 3.3 | Piličiauskas et al. 2017b |
| Lithuania | Coastal | Šventoji 6 | Subneolithic | Dog | -20.3 | 13.2 | 3.3 | Piličiauskas et al. 2017b |
| Poland | Inland | Dąbki 9 | Subneolithic | Pike | -24.0 | 10.4 | 3.5 | Robson et al. 2016 |
| Poland | Inland | Dąbki 9 | Subneolithic | Pike | -21.8 | 10.4 | 3.3 | Robson et al. 2016 |
| Poland | Inland | Dąbki 9 | Subneolithic | Pike | -24.2 | 8.6 | 3.5 | Robson et al. 2016 |
| Poland | Inland | Dąbki 9 | Subneolithic | Perch | -18.8 | 9.8 | 3.6 | Robson et al. 2016 |
| Poland | Inland | Dąbki 9 | Subneolithic | Perch | -17.9 | 9.1 | 3.3 | Robson et al. 2016 |
| Poland | Inland | Dąbki 9 | Subneolithic | Zander | -24.5 | 9.9 | 3.3 | Robson et al. 2016 |
| Poland | Inland | Dąbki 9 | Subneolithic | Zander | -21.6 | 11.8 | 3.3 | Robson et al. 2016 |
| Poland | Inland | Dąbki 9 | Subneolithic | Zander | -21.1 | 11.9 | 3.4 | Robson et al. 2016 |
| Russia | Inland | Sakhtysh Iia | Subneolithic | Human | -20.9 | 13.4 | 3.2 | Piezonka et al. 2013 |
| Russia | Inland | Sakhtysh Iia | Subneolithic | Human | -21.0 | 14.6 | 3.1 | Piezonka et al. 2013 |
| Russia | Inland | Sakhtysh Iia | Subneolithic | Human | -23.0 | 12.6 | 3.2 | Piezonka et al. 2013 |
| Russia | Inland | Sakhtysh Iia | Subneolithic | Human | -21.4 | 12.4 | 3.1 | Piezonka et al. 2013 |
| Lithuania | Coastal | Šventoji 6 | Subneolithic | Harp seal | -16.6 | 13.1 | 3.3 | Skipitytė unpublished data |
| Lithuania | Coastal | Šventoji 2/4 | Subneolithic | Grey seal | -16.7 | 11.6 | 3.4 | Skipitytė unpublished data |
| Lithuania | Coastal | Šventoji 23 | Subneolithic | Harbor seal | -15.9 | 12.0 | 3.2 | This study |
| Lithuania | Inland | Daktariškė 5 | Subneolithic | Human | -22.5 | 11.6 | 3.2 | This study |
| Lithuania | Inland | Daktariškė 5 | Subneolithic | Human | -21.2 | 12.8 | 3.2 | This study |
| Lithuania | Inland | Gyvakarai | Corded Ware | Human | -22.1 | 10.4 | 3.2 | Piličiauskas et al. 2017c |
| Lithuania | Inland | Plinkaigalis | Corded Ware | Human | -21.4 | 8.9 | 3.3 | Antanaitis-Jacobs et al. 2009 |
| Lithuania | Inland | Plinkaigalis | Corded Ware | Human | -21.5 | 9.8 | 3.3 | Antanaitis-Jacobs et al. 2009 |
| Lithuania | Inland | Benaičiai | Corded Ware | Human | -21.3 | 10.9 | 3.2 | Piličiauskas et al. 2017b |
| Lithuania | Inland | Biržai | Corded Ware | Human | -22.0 | 11.0 | 3.2 | Piličiauskas et al. 2017c |
| Lithuania | Inland | Biržai | Corded Ware | Human | -22.1 | 11.9 | 3.1 | Piličiauskas et al. 2017c |
| Lithuania | Inland | Biržai | Corded Ware | Human | -21.1 | 9.5 | 3.1 | Piličiauskas et al. 2017c |
| Lithuania | Inland | Biržai | Corded Ware | Human | -21.4 | 9.7 | 3.1 | Piličiauskas et al. 2017c |
| Lithuania | Inland | Benaičiai | Corded Ware | Human | -21.2 | 9.8 | 3.2 | Piličiauskas et al. 2017b |
| Latvia | Inland | Zvejnieki | Corded Ware | Human | -21.6 | 9.7 | 3.3 | Eriksson et al. 2003 |
| Latvia | Inland | Zvejnieki | Corded Ware | Human | -22.1 | 10.1 | 3.1 | Eriksson et al. 2003 |
| Latvia | Inland | Sarkani | Corded Ware | Human | -21.6 | 10.3 | 3.2 | Eriksson et al. 2003 |
| Latvia | Inland | Selgas | Corded Ware | Human | -21.3 | 10.4 | 3.2 | Eriksson et al. 2003 |
| Latvia | Inland | Selgas | Corded Ware | Human | -21.5 | 10.1 | 3.1 | Eriksson et al. 2003 |
| Latvia | Inland | Selgas | Corded Ware | Human | -21.8 | 11.8 | 3.2 | Eriksson et al. 2003 |
| Poland | Inland | Niedrzwica | Corded Ware | Human | -21.6 | 10.2 | 3.3 | Reitsema 2012 |
| Poland | Inland | Kruszyn | Corded Ware | Human | -21.9 | 10.2 |  | Pospieszny et al. 2015 |
| Sweden | Coastal | Lilla Bedinge | Corded Ware | Human | -20.4 | 9.0 | 3.5 | Fornander 2013 |
| Sweden | Coastal | Lilla Bedinge | Corded Ware | Human | -19.1 | 9.2 | 3.3 | Fornander 2013 |
| Sweden | Coastal | Lilla Bedinge | Corded Ware | Human | -21.3 | 8.8 | 3.3 | Fornander 2013 |
| Sweden | Coastal | Lilla Bedinge | Corded Ware | Human | -20.7 | 9.0 | 3.3 | Fornander 2013 |
| Sweden | Coastal | Lilla Bedinge | Corded Ware | Human | -20.9 | 9.2 | 3.3 | Fornander 2013 |
| Sweden | Coastal | Lilla Bedinge | Corded Ware | Human | -20.2 | 10.1 | 3.2 | Fornander 2013 |
| Sweden | Coastal | Lilla Bedinge | Corded Ware | Human | -20.1 | 10.7 | 3.3 | Fornander 2013 |
| Sweden | Coastal | Lilla Bedinge | Corded Ware | Human | -20.7 | 10.5 | 3.3 | Fornander 2013 |
| Sweden | Coastal | Lilla Bedinge | Corded Ware | Human | -20.3 | 10.2 | 3.3 | Fornander 2013 |
| Sweden | Coastal | Dösemarken | Corded Ware | Human | -20.9 | 8.6 | 3.2 | Fornander 2013 |
| Sweden | Coastal | Dösemarken | Corded Ware | Human | -18.7 | 10.8 | 3.3 | Fornander 2013 |
| Sweden | Coastal | Svågertorp | Corded Ware | Human | -20.2 | 9.4 | 3.3 | Fornander 2013 |
| Sweden | Coastal | Kastanjegården | Corded Ware | Human | -20.3 | 9.8 | 3.3 | Fornander 2013 |
| Sweden | Inland | Åraslöv | Corded Ware | Human | -18.8 | 12.4 | 3.3 | Fornander 2013 |
| Sweden | Inland | Håslöv | Corded Ware | Human | -19.1 | 11.8 | 3.2 | Fornander 2013 |
| Sweden | Inland | Håslöv | Corded Ware | Human | -20.2 | 11.3 | 3.3 | Fornander 2013 |
| Sweden | Inland | Håslöv | Corded Ware | Human | -20.8 | 9.8 | 3.2 | Fornander 2013 |
| Lithuania | Coastal | Benaičiai | Corded Ware | Human | -21.2 | 9.9 | 3.3 | Piličiauskas et al. 2017b |
| Lithuania | Coastal | Benaičiai | Corded Ware | Human | -21.4 | 10.6 | 3.3 | Piličiauskas et al. 2017b |
| Belarus | Inland | Krasnaselski | Corded Ware | Human | -21.2 | 10.9 | 3.3 | Piličiauskas et al. 2018a |
| Germany | Inland | Altdorf | Corded Ware | Human | -20.7 | 9.9 |  | Sjögren et al. 2016 |
| Germany | Inland | Bergrheinfeld | Corded Ware | Human | -19.5 | 11.1 |  | Sjögren et al. 2016 |
| Germany | Inland | Bergrheinfeld | Corded Ware | Human | -20.6 | 10.3 |  | Sjögren et al. 2016 |
| Germany | Inland | Bergrheinfeld | Corded Ware | Human | -19.5 | 9.7 |  | Sjögren et al. 2016 |
| Germany | Inland | Bergrheinfeld | Corded Ware | Human | -19.6 | 9.8 |  | Sjögren et al. 2016 |
| Germany | Inland | Bergrheinfeld | Corded Ware | Human | -19.2 | 10.0 |  | Sjögren et al. 2016 |
| Germany | Inland | Bergrheinfeld | Corded Ware | Human | -19.5 | 11.2 |  | Sjögren et al. 2016 |
| Germany | Inland | Bergrheinfeld | Corded Ware | Human | -20.5 | 9.9 |  | Sjögren et al. 2016 |
| Germany | Inland | Bergrheinfeld | Corded Ware | Human | -19.4 | 11.5 |  | Sjögren et al. 2016 |
| Germany | Inland | Bergrheinfeld | Corded Ware | Human | -19.3 | 11.3 |  | Sjögren et al. 2016 |
| Germany | Inland | Bergrheinfeld | Corded Ware | Human | -20.1 | 10.0 |  | Sjögren et al. 2016 |
| Germany | Inland | Bergrheinfeld | Corded Ware | Human | -19.7 | 11.3 |  | Sjögren et al. 2016 |
| Germany | Inland | Bergrheinfeld | Corded Ware | Human | -19.6 | 11.0 |  | Sjögren et al. 2016 |
| Germany | Inland | Bergrheinfeld | Corded Ware | Human | -19.5 | 11.1 |  | Sjögren et al. 2016 |
| Germany | Inland | Bergrheinfeld | Corded Ware | Human | -19.7 | 11.4 |  | Sjögren et al. 2016 |
| Germany | Inland | Bergrheinfeld | Corded Ware | Human | -19.3 | 10.5 |  | Sjögren et al. 2016 |
| Germany | Inland | Bergrheinfeld | Corded Ware | Human | -20.1 | 10.6 |  | Sjögren et al. 2016 |
| Germany | Inland | Bergrheinfeld | Corded Ware | Human | -19.3 | 9.2 |  | Sjögren et al. 2016 |
| Germany | Inland | Bergrheinfeld | Corded Ware | Human | -19.3 | 10.4 |  | Sjögren et al. 2016 |
| Germany | Inland | Kelheim | Corded Ware | Human | -20.6 | 10.2 |  | Sjögren et al. 2016 |
| Germany | Inland | Lauda-Königshofen | Corded Ware | Human | -19.6 | 11.3 |  | Sjögren et al. 2016 |
| Germany | Inland | Lauda-Königshofen | Corded Ware | Human | -19.8 | 11.8 |  | Sjögren et al. 2016 |
| Germany | Inland | Lauda-Königshofen | Corded Ware | Human | -19.7 | 11.0 |  | Sjögren et al. 2016 |
| Germany | Inland | Lauda-Königshofen | Corded Ware | Human | -19.7 | 11.2 |  | Sjögren et al. 2016 |
| Germany | Inland | Lauda-Königshofen | Corded Ware | Human | -19.6 | 11.5 |  | Sjögren et al. 2016 |
| Germany | Inland | Lauda-Königshofen | Corded Ware | Human | -19.7 | 11.5 |  | Sjögren et al. 2016 |
| Germany | Inland | Lauda-Königshofen | Corded Ware | Human | -20.0 | 12.0 |  | Sjögren et al. 2016 |
| Germany | Inland | Poldering | Corded Ware | Human | -21.0 | 9.8 |  | Sjögren et al. 2016 |
| Germany | Inland | Tiefbrunn | Corded Ware | Human | -21.4 | 11.3 |  | Sjögren et al. 2016 |
| Germany | Inland | Tiefbrunn | Corded Ware | Human | -21.3 | 11.2 |  | Sjögren et al. 2016 |
| Germany | Inland | Tiefbrunn | Corded Ware | Human | -21.5 | 10.8 |  | Sjögren et al. 2016 |
| Germany | Inland | Wolkshausen | Corded Ware | Human | -19.9 | 11.9 |  | Sjögren et al. 2016 |
| Poland | Inland | Kowal | Globular Amphora | Human | -20.3 | 9.9 | 3.2 | Reitsema 2012 |
| Poland | Inland | Koszyce | Globular Amphora | Human | -20.7 | 10.4 | 3.3 | Eriksson and Howcroft 2014 |
| Poland | Inland | Koszyce | Globular Amphora | Human | -21.5 | 9.9 | 3.3 | Eriksson and Howcroft 2014 |
| Poland | Inland | Koszyce | Globular Amphora | Human | -20.6 | 10.3 | 3.3 | Eriksson and Howcroft 2014 |
| Poland | Inland | Koszyce | Globular Amphora | Human | -20.2 | 10.3 | 3.3 | Eriksson and Howcroft 2014 |
| Poland | Inland | Koszyce | Globular Amphora | Human | -20.6 | 11.0 | 3.3 | Eriksson and Howcroft 2014 |
| Poland | Inland | Koszyce | Globular Amphora | Human | -20.4 | 10.4 | 3.3 | Eriksson and Howcroft 2014 |
| Poland | Inland | Koszyce | Globular Amphora | Human | -20.8 | 11.9 | 3.3 | Eriksson and Howcroft 2014 |
| Poland | Inland | Koszyce | Globular Amphora | Human | -20.5 | 11.2 | 3.3 | Eriksson and Howcroft 2014 |
| Poland | Inland | Koszyce | Globular Amphora | Human | -21.0 | 10.6 | 3.4 | Eriksson and Howcroft 2014 |
| Poland | Inland | Koszyce | Globular Amphora | Human | -20.6 | 10.9 | 3.2 | Eriksson and Howcroft 2014 |
| Poland | Inland | Koszyce | Globular Amphora | Human | -20.2 | 11.0 | 3.2 | Eriksson and Howcroft 2014 |
| Poland | Inland | Koszyce | Globular Amphora | Human | -20.3 | 10.4 | 3.2 | Eriksson and Howcroft 2014 |
| Poland | Inland | Koszyce | Globular Amphora | Human | -21.1 | 10.5 | 3.3 | Eriksson and Howcroft 2014 |
| Poland | Inland | Koszyce | Globular Amphora | Human | -20.2 | 10.6 | 3.2 | Eriksson and Howcroft 2014 |
| Poland | Inland | Koszyce | Globular Amphora | Human | -21.1 | 10.1 | 3.3 | Eriksson and Howcroft 2014 |
| Belarus | Inland | Krasnaselski | Globular Amphora | Cattle | -21.7 | 6.2 | 3.3 | Piličiauskas et al. 2018a |
| Lithuania | Coastal | Šventoji 41B | Post-Corded | Horse | -22.3 | 4.4 | 3.3 | Piličiauskas et al. 2017b |
| Lithuania | Coastal | Šventoji 9 | Post-Corded | Beaver | -22.9 | 4.5 | 3.2 | Piličiauskas et al. 2017b |
| Lithuania | Inland | Kretuonas 1D | Post-Corded | Auroch/bison | -22.3 | 3.1 | 3.3 | Antanaitis-Jacobs et al. 2009 |
| Sweden | Coastal | Torsborg subject VII (child) | Post-Corded | Human | -20.1 | 9.5 | 3.2 | Eriksson et al. 2008 |
| Sweden | Coastal | Vickleby subject I | Post-Corded | Human | -20.2 | 9.3 | 3.0 | Eriksson et al. 2008 |
| Sweden | Coastal | Vickleby subject I | Post-Corded | Human | -20.4 | 9.9 | 3.0 | Eriksson et al. 2008 |
| Sweden | Coastal | Algutsrum subject V | Post-Corded | Human | -19.7 | 10.2 | 3.2 | Eriksson et al. 2008 |
| Sweden | Coastal | Algutsrum subject VI | Post-Corded | Human | -20.4 | 8.9 | 3.0 | Eriksson et al. 2008 |
| Sweden | Coastal | Algutsrum subject VI | Post-Corded | Human | -19.5 | 9.2 | 3.0 | Eriksson et al. 2008 |
| Sweden | Coastal | Algutsrum subject VI | Post-Corded | Human | -19.9 | 9.1 | 3.2 | Eriksson et al. 2008 |
| Latvia | Inland | Zvejnieki | Post-Corded | Human | -21.3 | 11.0 | 3.2 | Eriksson et al. 2008 |
| Lithuania | Inland | Daktariškė 5 | Post-Corded | Pig | -21.7 | 4.5 | 3.3 | Piličiauskas et al. 2017c |
| Estonia | Coastal | Muuksi | Post-Corded | Human | -21.2 | 10.3 |  | Laneman and Lang 2013 |
| Estonia | Coastal | Muuksi | Post-Corded | Human | -21.1 | 10.2 |  | Laneman and Lang 2013 |
| Estonia | Coastal | Muuksi | Post-Corded | Human | -21.1 | 9.7 |  | Laneman and Lang 2013 |
| Estonia | Coastal | Muuksi | Post-Corded | Human | -21.3 | 10.4 |  | Laneman and Lang 2013 |
| Estonia | Coastal | Muuksi | Post-Corded | Human | -21.4 | 9.7 |  | Laneman and Lang 2013 |
| Estonia | Coastal | Muuksi | Post-Corded | Human | -21.2 | 9.6 |  | Laneman and Lang 2013 |
| Estonia | Coastal | Muuksi | Post-Corded | Human | -21.2 | 10.0 |  | Laneman and Lang 2013 |
| Estonia | Coastal | Muuksi | Post-Corded | Human | -21.2 | 11.2 |  | Laneman and Lang 2013 |
| Estonia | Inland | Kivisaare I | Post-Corded | Human | -22.0 | 11.7 | 3.4 | Tõrv and Meadows 2015 |
| Estonia | Coastal | Riigiküla | Post-Corded | Human | -21.2 | 10.6 | 3.5 | Tõrv and Meadows 2015 |
| Estonia | Coastal | Riigiküla | Post-Corded | Human | -21.9 | 10.7 | 3.4 | Tõrv and Meadows 2015 |
| Lithuania | Inland | Spiginas | Post-Corded | Human | -21.2 | 10.1 | 3.3 | Piličiauskas et al. 2017c |

***Table S2. Summary of samples analysed in this study showing the typological and sampling information as well as extraction procedure and analyses by GC-C-IRMS and EA-IRMS associated with each sherd. AE - acid extraction, SE - solvent extraction.***

| **Sample code** | **Ware** | **Vessel type** | **Sherd type** | **Sample type** | **Location** | **AE** | **SE** | **GC-C-IRMS** | **EA-IRMS** |
| --- | --- | --- | --- | --- | --- | --- | --- | --- | --- |
| ***Alksnynė 3*** | | | | | | |  |  |  |
| Al1 | Corded | Pot | Wall | Foodcrust | Interior | x |  |  | x |
| Al2 | Corded | Pot | Wall | Powder | Interior | x | x | x | x |
| Al3 | Corded | Pot | Wall | Foodcrust | Interior | x |  |  | x |
| Al4 | Corded | Pot | Wall | Foodcrust | Interior | x |  |  | x |
| Al5 | Corded | Pot | Wall | Sooted crust | Exterior | x |  |  | x |
| Al6 | Corded | Pot | Wall | Powder | Interior | x | x | x | x |
| Al7 | Corded | Pot | Base | Powder | Interior | x | x | x |  |
| Al8 | Corded | Beaker | Wall | Powder | Interior | x | x | x |  |
| Al9 | Corded | Pot | Wall | Powder | Interior | x |  |  |  |
| Al10 | Corded | Pot | Wall | Powder | Interior | x |  |  |  |
| ***Daktariškė 5*** | | | | | | |  |  |  |
| Dk623 | Post-Corded | Pot | Wall | Foodcrust | Interior | x |  | x | x |
| Dk417 | Post-Corded | Pot | Base | Foodcrust | Interior | x |  | x | x |
| DK494 | Post-Corded | Pot | Rim | Sooted crust | Exterior | x |  | x | x |
| Dk492 | Post-Corded | Pot | Wall | Foodcrust | Interior | x |  | x | x |
| Dk240 | Corded | Beaker | Rim | Foodcrust | Interior | x |  | x | x |
| DK342 | Post-Corded | Pot | Wall | Foodcrust | Interior | x |  | x | x |
| Dk525 | Corded | Pot | Wall | Foodcrust | Interior | x |  | x | x |
| Dk541 | Corded | Pot with cordon | Upper | Foodcrust | Interior | x |  | x | x |
| Dk524 | Post-Corded | Pot | Upper | Foodcrust | Interior | x |  | x | x |
| Dk323 | Corded | Pot | Wall | Foodcrust | Interior | x |  | x | x |
| DK87 I 4a | Hybrid | Pot | Neck | Sooted crust | Exterior | x |  | x | x |
| DK88 VIII 6a1 | Hybrid | Pot | Neck | Sooted crust | Exterior | x |  | x | x |
| DK87 III 3a1 | Corded | Amphora | Wall | Foodcrust | Interior | x |  | x | x |
| DK87 II 6a | Corded | Pot with cordon | Wall | Foodcrust | Interior | x |  | x | x |
| DK88 VII 5b | Corded | Pot with cordon | Rim | Foodcrust | Interior | x |  | x | x |
| DK87 V 6a3 | Hybrid | Pot | Rim | Foodcrust | Interior | x |  | x | x |
| DK87 I kontr. 1 | Globular Amphora | Pot | Rim | Foodcrust | Interior | x |  | x | x |
| DK87 ST | Globular Amphora | Pot | Neck | Sooted crust | Exterior | x |  | x | x |
| DK V 4a2 | Corded | Pot | Rim | Foodcrust | Interior | x |  | x | x |
| DK90 X 6b2 | Globular Amphora | Wide-mouth pot | Rim | Foodcrust | Interior | x |  |  | x |
| Dk466 | Corded | Beaker | Neck | Powder | Interior | x | x | x |  |
| Dk108 | Post-Corded | Pot | Wall | Powder | Interior | x |  |  |  |
| Dk482 | Post-Corded | Pot | Upper | Powder | Exterior/  interior | x |  |  |  |
| Dk419 | Post-Corded | Beaker | Neck | Powder | Interior | x |  |  |  |
| DK87 V 4b | Corded | Beaker | Rim | Foodcrust | Interior | x |  | x |  |
| Dk V 8a | Globular Amphora | Bowl | Rim | Powder | Interior | x | x | x |  |
| Dk X 8b1 | Globular Amphora | Pot | Rim | Foodcrust | Interior | x |  | x | x |
| ***Dubičiai 2*** | | | | | | | | | |
| Db2 perk. 7 | Corded | Beaker |  | Powder | Interior | x |  | x |  |
| Db2 perk. 8 | Corded | Beaker |  | Powder | Interior | x |  | x |  |
| ***Gribaša 4*** | | | | | | | | | |
| vessel No 3 | Globular Amphora | Amphora |  | Powder | Interior | x |  | x |  |
| vessel No 20 | Globular Amphora | Pot |  | Powder | Interior | x |  |  |  |
| ***Karaviškės 6*** | | | | | | | | | |
| H-32 | Corded | Beaker |  | Powder | Interior | x |  | x |  |
| EM2502:523 | Corded | Amphora |  | Powder | Interior | x |  |  |  |
| EM2502:533 | Corded | Beaker |  | Powder | Interior | x |  | x |  |
| EM2502:560 | Corded | Beaker |  | Powder | Interior | x |  | x |  |
| ***Kvietiniai*** | | | | | | |  |  |  |
| Kv1035 | Post-Corded | Pot | Wall | Foodcrust/powder | Interior | x |  | x | x |
| Kv2303 | Corded | Beaker | Wall | Powder | Interior | x |  | x | x |
| Kv732 | Post-Corded | Pot | Wall | Foodcrust/powder | Interior | x |  |  | x |
| Kv152 | Corded | Beaker | Wall | Powder | Interior | x | x | x | x |
| Kv1588 | Post-Corded | Pot | Wall | Powder | Interior | x |  | x | x |
| Kv228 | Post-Corded | Pot | Wall | Foodcrust | Interior | x |  | x | x |
| Kv2058 | Corded | Beaker | Neck | Powder | Interior | x |  | x |  |
| Kv1298 | Post-Corded | Pot | Neck | Powder | Interior | x | x | x |  |
| Kv1564 | Post-Corded | Pot | Rim | Foodcrust | Interior | x |  | x |  |
| Kv1349 | Corded | Beaker |  | Powder | Interior | x |  | x |  |
| Kv1895 | Corded | Beaker |  | Powder | Interior | x |  | x |  |
| Kv1878 | Corded | Beaker |  | Powder | Interior | x |  | x |  |
| ***Neravai*** | | | | | | | | | |
| Nr 14(38) | Corded | Beaker |  | Powder | Interior | x |  |  |  |
| ***Nida*** | | | | | | | | | |
| N78_3/4c | Rzucewo | Beaker |  | Powder | Interior | x |  | x |  |
| N75_I/9av | Rzucewo | Beaker |  | Powder | Interior | x |  | x |  |
| N75_I/22b | Rzucewo | Beaker |  | Powder | Interior | x |  |  |  |
| ***Šventoji 1*** | | | | | | | | | |
| Šv1-B14b | Corded | Pot | Base | Foodcrust | Interior | x |  | x | x |
| ***Šventoji 4*** | | | | | | |  |  |  |
| Šv4-2014-1059 | Globular Amphora | Amphora | Rim | Foodcrust | Interior | x |  | x | x |
| Šv4-2014-1029 | Globular Amphora | Pot | Rim | Foodcrust | Interior | x |  |  | x |

***Table S3. Bulk carbon (δ^13^C) and nitrogen (δ^15^N) stable isotope data obtained on carbonised surface residues. Note that some of the samples are replicated.***

| **Site** | **Location** | **Sample code** | **Ware** | **Vessel type** | **IRMS sample type** | **δ^13^C** | **δ^15^N** | **%C** | **%N** | **C:N**  **atomic ratio** | **Lab.** | **Year** | **Reference** |
| --- | --- | --- | --- | --- | --- | --- | --- | --- | --- | --- | --- | --- | --- |
| Alksnynė 3 | Lagoon | Al1 | Corded Ware | Pot | Foodcrust | -25.7 | 8.9 | 35.5 | 4.6 | 9.0 | Bradford | 2017 | This study |
| Alksnynė 3 | Lagoon | Al2 | Corded Ware | Pot | Sooted crust | -25.5 | 8.7 | 33.8 | 1.8 | 22.4 | Bradford | 2017 | This study |
| Alksnynė 3 | Lagoon | Al3 | Corded Ware | Pot | Foodcrust | -26.5 | 9.1 | 20.4 | 2.0 | 12.2 | Bradford | 2017 | This study |
| Alksnynė 3 | Lagoon | Al4 | Corded Ware | Pot | Foodcrust | -27.3 | 8.4 | 22.5 | 4.3 | 6.1 | Bradford | 2017 | This study |
| Alksnynė 3 | Lagoon | Al5 | Corded Ware | Pot | Sooted crust | -28.5 | 9.0 | 37.1 | 1.3 | 32.9 | Bradford | 2017 | This study |
| Alksnynė 3 | Lagoon | Al6 | Corded Ware | Pot | Foodcrust | -26.2 | 7.1 | 30.9 | 2.4 | 15.0 | Bradford | 2017 | This study |
| Daktariškė 1 | Inland | Dk1-9k | Globular Amphora | Pot | Sooted crust | -28.4 | 8.5 | 34.9 | 1.8 | 22.9 | Vilnius | 2017 | Piličiauskas et al. 2018b |
| Daktariškė 5 | Inland | Dk87 III 3a1 | Corded Ware | Amphora | Foodcrust | -26.9 | 4.3 | 52.2 | 4.0 | 15.2 | Vilnius | 2017 | This study |
| Daktariškė 5 | Inland | Dk87 III 1a | Corded Ware | Beaker | Sooted crust | -27.0 | 5.5 | 35.0 | 4.9 | 8.4 | Vilnius | 2017 | Piličiauskas et al. 2018b |
| Daktariškė 5 | Inland | Dk87 IV 5a1 | Corded Ware | Bowl | Foodcrust | -26.5 | 7.0 | 39.1 | 5.1 | 8.9 | Vilnius | 2017 | Piličiauskas et al. 2018b |
| Daktariškė 5 | Inland | Dk240 | Corded Ware | Pot | Foodcrust | -26.8 | 6.5 | 41.0 | 6.2 | 7.7 | Bradford | 2017 | This study |
| Daktariškė 5 | Inland | Dk525 | Corded Ware | Pot | Foodcrust | -27.1 | 7.3 | 39.0 | 5.9 | 7.7 | Bradford | 2017 | This study |
| Daktariškė 5 | Inland | Dk382 | Corded Ware | Pot | Foodcrust | -26.8 | 6.7 | 28.9 | 4.1 | 8.3 | Bradford | 2017 | Piličiauskas et al. 2018b |
| Daktariškė 5 | Inland | Dk90 | Corded Ware | Pot | Foodcrust | -27.8 | 7.7 | 37.0 | 4.5 | 9.6 | Bradford | 2017 | Piličiauskas et al. 2018b |
| Daktariškė 5 | Inland | Dk541 | Corded Ware | Pot | Foodcrust | -28.7 | 10.4 | 45.5 | 7.4 | 7.2 | Bradford | 2017 | This study |
| Daktariškė 5 | Inland | Dk323 | Corded Ware | Pot | Foodcrust | -25.5 | 4.5 | 43.4 | 4.1 | 12.3 | Bradford | 2017 | This study |
| Daktariškė 5 | Inland | Dk87 II 9b2 | Corded Ware | Pot | Foodcrust | -25.7 | 6.1 | 43.5 | 3.0 | 16.9 | Vilnius | 2017 | Piličiauskas et al. 2018b |
| Daktariškė 5 | Inland | Dk87 V 6a2 | Corded Ware | Pot | Foodcrust | -25.4 | 2.7 | 47.9 | 3.7 | 15.0 | Vilnius | 2017 | Piličiauskas et al. 2018b |
| Daktariškė 5 | Inland | Dk87 II 7a1 | Corded Ware | Pot | Foodcrust | -27.0 | 6.0 | 46.6 | 2.0 | 27.5 | Vilnius | 2017 | Piličiauskas et al. 2018b |
| Daktariškė 5 | Inland | Dk87 II 6a | Corded Ware | Pot | Foodcrust | -26.8 | 4.5 | 48.7 | 4.7 | 12.0 | Vilnius | 2017 | This study |
| Daktariškė 5 | Inland | Dk87 I 3a | Corded Ware | Pot | Foodcrust | -26.1 | 7.0 | 42.7 | 7.1 | 7.0 | Vilnius | 2017 | Piličiauskas et al. 2018b |
| Daktariškė 5 | Inland | Dk88 VII 5b | Corded Ware | Pot | Foodcrust | -25.7 | 5.3 | 49.6 | 4.3 | 13.5 | Vilnius | 2017 | This study |
| Daktariškė 5 | Inland | Dk88 VII 5a | Corded Ware | Pot | Sooted crust | -28.1 | 3.5 | 46.2 | 4.6 | 11.7 | Vilnius | 2017 | Piličiauskas et al. 2018b |
| Daktariškė 5 | Inland | Dk88 VIII 5b | Corded Ware | Pot | Foodcrust | -25.4 | 2.3 | 47.1 | 6.1 | 9.0 | Vilnius | 2017 | Piličiauskas et al. 2018b |
| Daktariškė 5 | Inland | Dk90 XI 6b | Corded Ware | Pot | Foodcrust | -26.6 | 2.0 | 52.5 | 4.2 | 14.6 | Vilnius | 2017 | Piličiauskas et al. 2018b |
| Daktariškė 5 | Inland | Dk90 XI 4b | Corded Ware | Pot | Foodcrust | -27.8 | 8.3 | 50.8 | 7.6 | 7.8 | Vilnius | 2017 | Piličiauskas et al. 2018b |
| Daktariškė 5 | Inland | Dk IV 5a2 | Corded Ware | Pot | Foodcrust | -27.8 | 3.2 | 47.2 | 4.7 | 11.8 | Vilnius | 2017 | Piličiauskas et al. 2018b |
| Daktariškė 5 | Inland | Dk IV 5a2 | Corded Ware | Pot | Foodcrust | -27.6 | 3.1 | 48.6 | 4.6 | 12.3 | Bradford | 2014 | Piličiauskas et al. 2018b |
| Daktariškė 5 | Inland | Dk IV 4b | Corded Ware | Pot | Foodcrust | -26.5 | 4.4 | 45.2 | 4.7 | 11.2 | Vilnius | 2017 | Piličiauskas et al. 2018b |
| Daktariškė 5 | Inland | Dk V 4a2 | Corded Ware | Pot | Foodcrust | -26.4 | 4.5 | 54.9 | 3.9 | 16.3 | Vilnius | 2017 | This study |
| Daktariškė 5 | Inland | Dk EM2245:2228 | Corded Ware | Pot | Foodcrust | -29.2 | 5.2 | 13.4 | 1.0 | 14.9 | Vilnius | 2017 | Piličiauskas et al. 2018b |
| Daktariškė 5 | Inland | Dk V 4b | Corded Ware | Pot | Foodcrust | -26.9 | 5.1 | 46.6 | 5.6 | 9.7 | Bradford | 2014 | Piličiauskas et al. 2018b |
| Daktariškė 5 | Inland | Dk VI 1b | Corded Ware | Pot | Foodcrust | -27.1 | 6.2 | 24.7 | 3.6 | 8.0 | Vilnius | 2017 | Piličiauskas et al. 2018b |
| Daktariškė 5 | Inland | Dk X 8b2 | Corded Ware | Pot | Foodcrust | -26.7 | 6.2 | 49.2 | 4.2 | 13.8 | Vilnius | 2017 | Piličiauskas et al. 2018b |
| Daktariškė 5 | Inland | Dk87 I kontr. 2 | Corded Ware | Pot | Foodcrust | -27.1 | 5.6 | 52.4 | 8.8 | 7.0 | Vilnius | 2017 | Piličiauskas et al. 2018b |
| Daktariškė 5 | Inland | - | Corded Ware | Pot | Foodcrust | -26.2 | 3.5 | 47.3 | 3.7 | 14.8 | Bradford | 2014 | Piličiauskas et al. 2018b |
| Daktariškė 5 | Inland | Dk87 V 6a1 | Globular Amphora | Amphora | Foodcrust | -24.6 | 4.4 | 56.8 | 2.1 | 31.2 | Vilnius | 2017 | Piličiauskas et al. 2018b |
| Daktariškė 5 | Inland | Dk X 8b1 | Globular Amphora | Pot | Foodcrust | -27.0 | 5.9 | 52.9 | 5.1 | 12.2 | Vilnius | 2017 | This study |
| Daktariškė 5 | Inland | Dk87 I kontr. 1 | Globular Amphora | Pot | Foodcrust | -28.8 | 6.2 | 39.0 | 6.5 | 7.0 | Vilnius | 2017 | This study |
| Daktariškė 5 | Inland | X 8b1 | Globular Amphora | Pot | Foodcrust | -26.8 | 6.8 | 53.6 | 6.4 | 9.8 | Bradford | 2014 | Piličiauskas et al. 2018b |
| Daktariškė 5 | Inland | Dk87 | Globular Amphora | Pot | Foodcrust | -28.1 | 7.0 | 45.0 | 5.7 | 9.2 | Bradford | 2014 | Piličiauskas et al. 2018b |
| Daktariškė 5 | Inland | Dk VIII | Globular Amphora | Pot | Foodcrust | -29.4 | 7.2 | 46.9 | 7.1 | 7.7 | Vilnius | 2017 | Piličiauskas et al. 2018b |
| Daktariškė 5 | Inland | Dk 87FD | Globular Amphora | Pot | Foodcrust | -28.9 | 7.4 | 54.0 | 6.6 | 9.6 | Vilnius | 2017 | Piličiauskas et al. 2018b |
| Daktariškė 5 | Inland | Dk VIII 6a2 | Globular Amphora | Amphora | Foodcrust | -27.7 | 7.5 | 48.3 | 6.4 | 8.7 | Vilnius | 2017 | Piličiauskas et al. 2018b |
| Daktariškė 5 | Inland | Dk90 X 6b2 | Globular Amphora | Pot | Foodcrust | -28.7 | 8.7 | 52.7 | 8.2 | 7.5 | Vilnius | 2017 | This study |
| Daktariškė 5 | Inland | Dk 87ST | Globular Amphora | Pot | Sooted crust | -26.8 | 9.1 | 51.3 | 6.8 | 8.8 | Vilnius | 2017 | This study |
| Daktariškė 5 | Inland | Dk90 EM2245:2235 | Globular Amphora | Pot | Sooted crust | -25.3 | 14.3 | 44.5 | 4.5 | 11.6 | Vilnius | 2017 | Piličiauskas et al. 2018b |
| Daktariškė 5 | Inland | Dk16-329B | Hybrid Ware | Pot | Foodcrust | -27.8 | 7.9 | 45.7 | 5.0 | 10.6 | Vilnius | 2017 | Piličiauskas et al. 2018b |
| Daktariškė 5 | Inland | Dk87 III 2b | Hybrid Ware | Pot | Foodcrust | -26.4 | 6.0 | 31.6 | 5.3 | 7.0 | Vilnius | 2017 | Piličiauskas et al. 2018b |
| Daktariškė 5 | Inland | Dk87 III 2a | Hybrid Ware | Pot | Foodcrust | -26.5 | 10.3 | 48.8 | 3.6 | 15.8 | Vilnius | 2017 | Piličiauskas et al. 2018b |
| Daktariškė 5 | Inland | Dk87 I 4a | Hybrid Ware | Pot | Sooted crust | -26.2 | 8.9 | 40.7 | 2.2 | 21.8 | Vilnius | 2017 | This study |
| Daktariškė 5 | Inland | Dk88 VIII 6a1 | Hybrid Ware | Pot | Sooted crust | -26.5 | 9.9 | 51.6 | 5.2 | 11.5 | Vilnius | 2017 | This study |
| Daktariškė 5 | Inland | Dk87 III 4b | Hybrid Ware | Pot | Sooted crust | -25.7 | 9.6 | 50.4 | 5.1 | 11.6 | Vilnius | 2017 | Piličiauskas et al. 2018b |
| Daktariškė 5 | Inland | Dk87 V 6a3 | Hybrid Ware | Pot | Foodcrust | -28.5 | 9.8 | 49.6 | 6.1 | 9.4 | Vilnius | 2017 | This study |
| Daktariškė 5 | Inland | Dk87 I 5b | Hybrid Ware | Pot | Sooted crust | -27.1 | 8.2 | 53.5 | 3.8 | 16.3 | Vilnius | 2017 | Piličiauskas et al. 2018b |
| Daktariškė 5 | Inland | Dk V 3a | Hybrid Ware | Pot | Sooted crust | -26.5 | 9.4 | 47.1 | 4.8 | 11.4 | Vilnius | 2017 | Piličiauskas et al. 2018b |
| Daktariškė 5 | Inland | Dk III 3a | Hybrid Ware | Pot | Foodcrust | -26.4 | 7.9 | 49.6 | 7.0 | 8.3 | Vilnius | 2017 | Piličiauskas et al. 2018b |
| Daktariškė 5 | Inland | Dk VI 5b | Hybrid Ware | Pot | Sooted crust | -26.4 | 10.3 | 54.3 | 4.2 | 15.2 | Vilnius | 2017 | Piličiauskas et al. 2018b |
| Daktariškė 5 | Inland | Dk EM2245:574 | Hybrid Ware | Pot | Foodcrust | -27.3 | 6.2 | 40.5 | 4.3 | 11.1 | Vilnius | 2017 | Piličiauskas et al. 2018b |
| Daktariškė 5 | Inland | Dk16-279Bv | Porous Ware | Pot | Sooted crust | -28.5 | 9.2 | 45.9 | 6.4 | 8.4 | Vilnius | 2017 | Piličiauskas et al. 2018b |
| Daktariškė 5 | Inland | Dk16-484B4 | Porous Ware | Pot | Sooted crust | -26.9 | 11.4 | 53.5 | 5.9 | 10.5 | Vilnius | 2017 | Piličiauskas et al. 2018b |
| Daktariškė 5 | Inland | Dk II 8b | Porous Ware | Pot | Sooted crust | -26.5 | 11.9 | 50.2 | 6.0 | 9.8 | Vilnius | 2017 | Piličiauskas et al. 2018b |
| Daktariškė 5 | Inland | Dk I 6a | Porous Ware | Pot | Sooted crust | -27.1 | 11.4 | 50.4 | 4.8 | 12.3 | Vilnius | 2017 | Piličiauskas et al. 2018b |
| Daktariškė 5 | Inland | Dk II 9a1 | Porous Ware | Pot | Sooted crust | -27.9 | 9.4 | 52.1 | 5.3 | 11.6 | Vilnius | 2017 | Piličiauskas et al. 2018b |
| Daktariškė 5 | Inland | Dk II 9a2 | Porous Ware | Pot | Foodcrust | -29.3 | 8.6 | 46.8 | 6.8 | 8.0 | Vilnius | 2017 | Piličiauskas et al. 2018b |
| Daktariškė 5 | Inland | II 8a | Porous Ware | Pot | Foodcrust | -28.6 | 9.5 | 43.7 | 6.8 | 7.5 | Bradford | 2014 | Piličiauskas et al. 2018b |
| Daktariškė 5 | Inland | Dk623 | Post-Corded Ware | Pot | Foodcrust | -26.6 | 6.1 | 51.6 | 6.4 | 9.4 | Bradford | 2017 | This study |
| Daktariškė 5 | Inland | Dk438 | Post-Corded Ware | Pot | Foodcrust | -28.2 | 3.3 | 39.1 | 3.6 | 12.8 | Bradford | 2017 | Piličiauskas et al. 2018b |
| Daktariškė 5 | Inland | Dk417 | Post-Corded Ware | Pot | Sooted crust | -25.5 | 6.2 | 33.1 | 2.8 | 13.8 | Bradford | 2017 | This study |
| Daktariškė 5 | Inland | Dk59 | Post-Corded Ware | Pot | Foodcrust | -27.1 | 7.6 | 43.0 | 5.6 | 8.9 | Bradford | 2017 | Piličiauskas et al. 2018b |
| Daktariškė 5 | Inland | Dk268 | Post-Corded Ware | Pot | Foodcrust | -25.7 | 6.1 | 47.5 | 3.7 | 15.0 | Bradford | 2017 | Piličiauskas et al. 2018b |
| Daktariškė 5 | Inland | Dk128 | Post-Corded Ware | Pot | Foodcrust | -27.0 | 3.4 | 28.0 | 2.1 | 15.3 | Bradford | 2017 | Piličiauskas et al. 2018b |
| Daktariškė 5 | Inland | Dk433 | Post-Corded Ware | Pot | Foodcrust | -26.0 | 7.9 | 36.7 | 6.7 | 6.4 | Bradford | 2017 | Piličiauskas et al. 2018b |
| Daktariškė 5 | Inland | Dk207 | Post-Corded Ware | Pot | Foodcrust | -27.2 | 5.4 | 41.9 | 5.5 | 8.9 | Bradford | 2017 | Piličiauskas et al. 2018b |
| Daktariškė 5 | Inland | Dk376 | Post-Corded Ware | Pot | Foodcrust | -26.9 | 5.2 | 45.9 | 5.6 | 9.6 | Bradford | 2017 | Piličiauskas et al. 2018b |
| Daktariškė 5 | Inland | Dk494 | Post-Corded Ware | Pot | Sooted crust | -26.3 | 4.6 | 57.0 | 5.5 | 12.2 | Bradford | 2017 | This study |
| Daktariškė 5 | Inland | Dk492 | Post-corded Ware | Pot | Foodcrust | -27.9 | 8.6 | 29.4 | 4.2 | 8.1 | Bradford | 2017 | This study |
| Daktariškė 5 | Inland | Dk492 | Post-Corded Ware | Pot | Sooted crust | -26.2 | 10.8 | 48.4 | 2.9 | 19.6 | Bradford | 2017 | This study |
| Daktariškė 5 | Inland | Dk342 | Post-Corded Ware | Pot | Foodcrust | -25.7 | 2.3 | 51.9 | 4.1 | 14.6 | Bradford | 2017 | This study |
| Daktariškė 5 | Inland | Dk524 | Post-Corded Ware | Pot | Foodcrust | -25.8 | 9.4 | 47.1 | 6.3 | 8.7 | Bradford | 2017 | This study |
| Daktariškė 5 | Inland | Dk87 V 4a1 | Post-Corded Ware | Pot | Sooted crust | -26.1 | 8.1 | 50.2 | 3.6 | 16.2 | Vilnius | 2017 | Piličiauskas et al. 2018b |
| Daktariškė 5 | Inland | Dk87 III 1b | Post-Corded Ware | Pot | Foodcrust | -27.8 | 5.6 | 44.2 | 6.0 | 8.6 | Vilnius | 2017 | Piličiauskas et al. 2018b |
| Daktariškė 5 | Inland | Dk87 II 9b1 | Post-Corded Ware | Pot | Foodcrust | -26.9 | 7.0 | 54.6 | 5.8 | 11.0 | Vilnius | 2017 | Piličiauskas et al. 2018b |
| Daktariškė 5 | Inland | Dk87 I 7a | Post-Corded Ware | Pot | Sooted crust | -26.0 | 9.7 | 50.8 | 6.7 | 8.8 | Vilnius | 2017 | Piličiauskas et al. 2018b |
| Daktariškė 5 | Inland | Dk87 I 6b2 | Post-Corded Ware | Pot | Sooted crust | -26.4 | 7.2 | 40.6 | 4.4 | 10.7 | Vilnius | 2017 | Piličiauskas et al. 2018b |
| Daktariškė 5 | Inland | Dk89 X 4b | Post-Corded Ware | Pot | Foodcrust | -26.6 | 7.3 | 45.4 | 5.0 | 10.5 | Vilnius | 2017 | Piličiauskas et al. 2018b |
| Daktariškė 5 | Inland | Dk87 I 5a | Post-Corded Ware | Pot | Sooted crust |  | 3.6 | 36.1 |  | 0.0 | Vilnius | 2017 | Piličiauskas et al. 2018b |
| Daktariškė 5 | Inland | Dk87 I/II | Post-Corded Ware | Pot | Foodcrust | -25.0 | 3.5 | 34.6 | 2.2 | 18.4 | Vilnius | 2017 | Piličiauskas et al. 2018b |
| Daktariškė 5 | Inland | Dk II 7a2 | Post-Corded Ware | Pot | Sooted crust | -26.7 | 9.4 | 44.4 | 7.0 | 7.5 | Vilnius | 2017 | Piličiauskas et al. 2018b |
| Daktariškė 5 | Inland | Dk III | Post-Corded Ware | Pot | Foodcrust | -25.5 | 5.1 | 44.8 | 4.8 | 10.8 | Vilnius | 2017 | Piličiauskas et al. 2018b |
| Daktariškė 5 | Inland | Dk EM2245:950 | Post-Corded Ware | Pot | Foodcrust | -26.7 | 7.1 | 46.2 | 5.6 | 9.6 | Bradford | 2014 | Piličiauskas et al. 2018b |
| Daktariškė 5 | Inland | Dk EM2245:950 | Post-Corded Ware | Pot | Foodcrust | -27.0 | 6.9 | 51.1 | 6.3 | 9.4 | Vilnius | 2017 | Piličiauskas et al. 2018b |
| Daktariškė 5 | Inland | Dk III 6b | Post-Corded Ware | Pot | Sooted crust | -26.4 | 10.7 | 53.6 | 9.4 | 6.7 | Vilnius | 2017 | Piličiauskas et al. 2018b |
| Daktariškė 5 | Inland | Dk IX 3b | Post-Corded Ware | Pot | Foodcrust | -26.6 | 6.2 | 43.5 | 4.1 | 12.4 | Vilnius | 2017 | Piličiauskas et al. 2018b |
| Gaigalinė 1 | Inland | Ga-I2y | Corded Ware | Pot | Foodcrust | -27.6 | 2.1 | 43.5 | 2.3 | 21.8 | Vilnius | 2017 | Piličiauskas et al. 2018b |
| Gaigalinė 2 | Inland | Ga2-III 9n(1) | Corded Ware | Pot | Foodcrust | -25.9 | 3.2 | 26.7 | 1.5 | 21.4 | Vilnius | 2017 | Piličiauskas et al. 2018b |
| Gaigalinė 2 | Inland | Ga2-III 9n(2) | Corded Ware | Pot | Foodcrust | -25.5 | 3.4 | 23.8 | 1.2 | 23.2 | Vilnius | 2017 | Piličiauskas et al. 2018b |
| Karaviškės 6 | Inland | Kr05-2777 | Corded Ware | Pot | Foodcrust | -26.2 | 2.4 | 38.0 | 1.5 | 29.6 | Vilnius | 2017 | Piličiauskas et al. 2018b |
| Katros ištakos 1 | Inland | Kt-480 | Corded Ware | Pot | Foodcrust | -27.6 | 5.9 | 23.0 | 1.8 | 15.3 | Vilnius | 2017 | Piličiauskas et al. 2018b |
| Katros ištakos 1 | Inland | Kt-260 | Corded Ware | Pot | Foodcrust | -27.1 | 3.6 | 26.2 | 1.4 | 22.1 | Vilnius | 2017 | Piličiauskas et al. 2018b |
| Kunigiškiai | Inland | Kng-Š1 | Corded Ware | Pot | Foodcrust | -27.6 | 2.7 | 36.2 | 1.9 | 22.2 | Vilnius | 2017 | Piličiauskas et al. 2018b |
| Kvietiniai | Inland | Kv2303 | Corded Ware | Beaker | Foodcrust | -27.4 | 5.6 | 44.1 | 3.5 | 14.7 | Bradford | 2017 | This study |
| Kvietiniai | Inland | Kv152 | Corded Ware | Pot | Foodcrust | -26.3 | 6.2 | 36.0 | 2.2 | 18.9 | Bradford | 2017 | This study |
| Kvietiniai | Inland | Kv1035 | Post-Corded Ware | Pot | Foodcrust | -27.0 | 5.5 | 43.3 | 5.2 | 9.8 | Bradford | 2017 | This study |
| Kvietiniai | Inland | Kv732 | Post-Corded Ware | Pot | Foodcrust | -26.0 | 5.1 | 39.2 | 5.2 | 8.8 | Bradford | 2017 | This study |
| Kvietiniai | Inland | Kv1588 | Post-Corded Ware | Pot | Foodcrust | -27.6 | 7.2 | 37.3 | 3.5 | 12.5 | Bradford | 2017 | This study |
| Kvietiniai | Inland | Kv228 | Post-Corded Ware | Pot | Foodcrust | -27.0 | 7.5 | 44.7 | 3.0 | 17.3 | Bradford | 2017 | This study |
| Margiai 1 | Inland | Mrg1-EM2258:958 | Corded Ware | Pot | Foodcrust | -27.2 | 5.3 | 37.2 | 2.4 | 18.1 | Vilnius | 2017 | Piličiauskas et al. 2018b |
| Margiai 1 | Inland | Mrg1-1980slA | Corded Ware | Pot | Foodcrust | -26.8 | 5.0 | 34.6 | 3.0 | 13.5 | Vilnius | 2017 | Piličiauskas et al. 2018b |
| Margiai 1 | Inland | Mrg1-7a | Corded Ware | Pot | Foodcrust | -25.8 | 2.7 | 28.4 | 2.0 | 16.7 | Vilnius | 2017 | Piličiauskas et al. 2018b |
| Margiai 1 | Inland | Mrg1-B/16p | Corded Ware | Pot | Foodcrust | -27.0 | 2.9 | 41.6 | 2.2 | 21.7 | Vilnius | 2017 | Piličiauskas et al. 2018b |
| Nida | Lagoon | Nd9366 | Porous Ware | Pot | Sooted crust | -29.4 | 10.6 | 38.9 | 5.3 | 8.6 | Bradford | 2017 | Piličiauskas et al. 2018b |
| Nida | Lagoon | Nd9953 | Porous Ware | Pot | Foodcrust | -28.7 | 10.1 | 26.6 | 3.9 | 7.9 | Bradford | 2017 | Piličiauskas et al. 2018b |
| Nida | Lagoon | - | Porous Ware | Pot | Foodcrust | -28.8 | 9.8 | 30.2 | 6.1 | 5.7 | Bradford | 2014 | Piličiauskas et al. 2018b |
| Papiškės 4 | Inland | Pap4-Š3 | Porous Ware | Pot | Foodcrust | -27.0 | 7.5 | 43.2 | 4.9 | 10.2 | Vilnius | 2017 | Piličiauskas et al. 2018b |
| Šarnelė | Inland | Šr82 | Corded Ware | Pot | Foodcrust | -26.8 | 5.3 | 33.2 | 5.5 | 7.0 | Vilnius | 2017 | Piličiauskas et al. 2018b |
| Šarnelė | Inland | - | Corded Ware | Pot | Foodcrust | -26.6 | 5.7 | 36.7 | 6.2 | 6.9 | Bradford | 2014 | Piličiauskas et al. 2018b |
| Šarnelė | Inland | EM2237:36 | Corded Ware | Pot | Foodcrust | -28.6 | 7.7 | 34.3 | 4.6 | 8.8 | Bradford | 2014 | Piličiauskas et al. 2018b |
| Šarnelė | Inland | Šr82 II EM2237:70 | Hybrid Ware | Pot | Sooted crust | -27.9 | 8.1 | 39.6 | 4.5 | 10.3 | Vilnius | 2017 | Piličiauskas et al. 2018b |
| Šarnelė | Inland | 9b | Post-Corded Ware | Pot | Foodcrust | -25.5 | 2.0 | 54.6 | 3.4 | 18.9 | Bradford | 2014 | Piličiauskas et al. 2018b |
| Šarnelė | Inland | Šr73 EM2273:29(2) | Post-Corded Ware | Pot | Sooted crust | -27.3 | 8.4 | 52.8 | 4.8 | 12.8 | Vilnius | 2017 | Piličiauskas et al. 2018b |
| Šarnelė | Inland | Šr73 EM2273:29 | Post-Corded Ware | Pot | Foodcrust | -26.1 | 4.4 | 37.7 | 3.5 | 13.2 | Bradford | 2014 | Piličiauskas et al. 2018b |
| Šarnelė | Inland | EM2237:34 | Post-Corded Ware | Pot | Sooted crust | -27.2 | 8.9 | 40.6 | 2.6 | 18.1 | Vilnius | 2017 | Piličiauskas et al. 2018b |
| Skirmantinė 1 | Inland | Sk1-9k | Corded Ware | Beaker | Foodcrust | -25.5 | 6.7 | 41.4 | 3.9 | 12.2 | Vilnius | 2017 | Piličiauskas et al. 2018b |
| Šventoji 1 | Lagoon | Šv1-A12a(2) | Corded Ware | Beaker | Foodcrust | -26.5 | 3.9 | 55.0 | 4.7 | 13.6 | Vilnius | 2017 | Piličiauskas et al. 2018b |
| Šventoji 1 | Lagoon | Šv1-A6c | Corded Ware | Pot | Foodcrust | -28.0 | 6.2 | 53.7 | 4.4 | 14.2 | Vilnius | 2017 | Piličiauskas et al. 2018b |
| Šventoji 1 | Lagoon | Šv1-A7c | Corded Ware | Pot | Foodcrust | -26.1 | 4.0 | 56.3 | 4.3 | 15.4 | Vilnius | 2017 | Piličiauskas et al. 2018b |
| Šventoji 1 | Lagoon | Šv1-A12a | Corded Ware | Pot | Foodcrust | -25.9 | 3.6 | 50.7 | 2.7 | 22.2 | Vilnius | 2017 | Piličiauskas et al. 2018b |
| Šventoji 1 | Lagoon | Šv1-A7g | Corded Ware | Pot | Foodcrust | -24.5 | 9.5 | 46.5 | 5.6 | 9.7 | Vilnius | 2017 | Piličiauskas et al. 2018b |
| Šventoji 1 | Lagoon | Šv1-A35i | Corded Ware | Pot | Foodcrust | -27.5 | 6.4 | 52.3 | 4.5 | 13.4 | Vilnius | 2017 | Piličiauskas et al. 2018b |
| Šventoji 1 | Lagoon | Šv1-A13-14/a-c(2) | Corded Ware | Pot | Foodcrust | -26.5 | 10.3 | 53.0 | 6.0 | 10.4 | Vilnius | 2017 | Piličiauskas et al. 2018b |
| Šventoji 1 | Lagoon | Šv1-B14b | Corded Ware | Pot | Foodcrust | -26.2 | 5.4 | 46.8 | 5.3 | 10.3 | Vilnius | 2017 | This study |
| Šventoji 1 | Lagoon | Šv1-A35g | Globular Amphora | Pot | Foodcrust | -27.3 | 2.1 | 52.5 | 3.5 | 17.3 | Vilnius | 2017 | Piličiauskas et al. 2018b |
| Šventoji 1 | Lagoon | Šv1-A9c | Globular Amphora | Pot | Foodcrust | -27.7 | 6.6 | 46.7 | 5.1 | 10.7 | Vilnius | 2017 | Piličiauskas et al. 2018b |
| Šventoji 1 | Lagoon | Šv1-A33g(2) | Globular Amphora | Pot | Foodcrust | -29.2 | 6.8 | 50.0 | 4.3 | 13.5 | Vilnius | 2017 | Piličiauskas et al. 2018b |
| Šventoji 1 | Lagoon | Šv1-A12c | Globular Amphora | Pot | Sooted crust | -26.0 | 6.8 | 40.4 | 3.0 | 15.6 | Vilnius | 2017 | Piličiauskas et al. 2018b |
| Šventoji 1 | Lagoon | Šv1-A9b | Globular Amphora | Pot | Foodcrust | -27.3 | 6.9 | 44.7 | 2.8 | 18.8 | Vilnius | 2017 | Piličiauskas et al. 2018b |
| Šventoji 1 | Lagoon | Šv1-A26a | Globular Amphora | Bowl | Foodcrust | -27.8 | 6.9 | 46.6 | 6.8 | 8.0 | Vilnius | 2017 | Piličiauskas et al. 2018b |
| Šventoji 1 | Lagoon | Šv1-A2a-c(2) | Globular Amphora | Pot | Foodcrust | -25.8 | 7.5 | 51.8 | 4.4 | 13.8 | Vilnius | 2017 | Piličiauskas et al. 2018b |
| Šventoji 1 | Lagoon | Šv1-A9c(4) | Globular Amphora | Pot | Sooted crust | -28.2 | 7.9 | 55.7 | 5.9 | 11.0 | Vilnius | 2017 | Piličiauskas et al. 2018b |
| Šventoji 1 | Lagoon | Šv1-A9d | Globular Amphora | Pot | Sooted crust | -27.4 | 8.0 | 50.3 | 3.7 | 16.0 | Vilnius | 2017 | Piličiauskas et al. 2018b |
| Šventoji 1 | Lagoon | Šv1-A9b(2) | Globular Amphora | Pot | Sooted crust | -27.3 | 8.1 | 50.5 | 5.0 | 11.8 | Vilnius | 2017 | Piličiauskas et al. 2018b |
| Šventoji 1 | Lagoon | Šv1-A13-14/a-c | Globular Amphora | Pot | Foodcrust | -28.5 | 8.2 | 60.1 | 5.9 | 11.9 | Vilnius | 2017 | Piličiauskas et al. 2018b |
| Šventoji 1 | Lagoon | Šv1-A1b | Globular Amphora | Pot | Sooted crust | -25.8 | 8.5 | 55.5 | 5.7 | 11.4 | Vilnius | 2017 | Piličiauskas et al. 2018b |
| Šventoji 1 | Lagoon | Šv1-B8c+A7c | Globular Amphora | Pot | Foodcrust | -27.5 | 8.5 | 49.0 | 7.7 | 7.4 | Vilnius | 2017 | Piličiauskas et al. 2018b |
| Šventoji 1 | Lagoon | Šv1-A4c | Globular Amphora | Pot | Foodcrust | -26.8 | 8.8 | 44.6 | 5.5 | 9.5 | Vilnius | 2017 | Piličiauskas et al. 2018b |
| Šventoji 1 | Lagoon | Šv1-A14a | Globular Amphora | Pot | Foodcrust | -27.4 | 9.1 | 61.7 | 6.3 | 11.3 | Vilnius | 2017 | Piličiauskas et al. 2018b |
| Šventoji 1 | Lagoon | Šv1-A33ė | Globular Amphora | Pot | Foodcrust | -26.5 | 9.5 | 53.0 | 5.4 | 11.4 | Vilnius | 2017 | Piličiauskas et al. 2018b |
| Šventoji 1 | Lagoon | Šv1-A2a-c | Globular Amphora | Pot | Sooted crust | -28.9 | 9.6 | 47.2 | 5.8 | 9.4 | Vilnius | 2017 | Piličiauskas et al. 2018b |
| Šventoji 1 | Lagoon | Šv1-A9c(3) | Globular Amphora | Pot | Foodcrust | -30.8 | 9.8 | 43.4 | 5.4 | 9.3 | Vilnius | 2017 | Piličiauskas et al. 2018b |
| Šventoji 1 | Lagoon | Šv1-2006-97š | Globular Amphora | Bowl | Foodcrust | -26.1 | 9.9 | 50.1 | 8.2 | 7.1 | Vilnius | 2017 | Piličiauskas et al. 2018b |
| Šventoji 1 | Lagoon | 95 testpit | Globular Amphora | Pot | Foodcrust | -26.8 | 10.0 | 42.0 | 6.2 | 7.9 | Bradford | 2014 | Piličiauskas et al. 2018b |
| Šventoji 1 | Lagoon | Šv1-A7c(2) | Globular Amphora | Pot | Sooted crust | -25.6 | 10.4 | 56.3 | 5.0 | 13.3 | Vilnius | 2017 | Piličiauskas et al. 2018b |
| Šventoji 1 | Lagoon | Šv1-A9c(2) | Globular Amphora | Pot | Foodcrust | -25.2 | 11.2 | 56.9 | 5.1 | 13.1 | Vilnius | 2017 | Piličiauskas et al. 2018b |
| Šventoji 1 | Lagoon | Šv1-A1a | Globular Amphora | Pot | Foodcrust | -24.4 | 12.3 | 50.2 | 8.4 | 7.0 | Vilnius | 2017 | Piličiauskas et al. 2018b |
| Šventoji 1 | Lagoon | Šv1-EM2070:114 | Hybrid Ware | Pot | Foodcrust | -25.3 | 9.4 | 58.6 | 7.9 | 8.6 | Vilnius | 2017 | Piličiauskas et al. 2018b |
| Šventoji 2 | Lagoon | Šv2-A3a | Globular Amphora | Pot | Sooted crust | -27.7 | 8.8 | 40.2 | 6.1 | 7.6 | Vilnius | 2017 | Piličiauskas et al. 2018b |
| Šventoji 2 | Lagoon | Šv2-EM2135:39 | Globular Amphora | Pot | Foodcrust | -26.4 | 9.7 | 44.4 | 3.6 | 14.3 | Vilnius | 2017 | Piličiauskas et al. 2018b |
| Šventoji 2 | Lagoon | EM2135:141 | Globular Amphora | Pot | Sooted crust | -26.1 | 9.8 | 49.1 | 3.2 | 18.1 | Bradford | 2014 | Piličiauskas et al. 2018b |
| Šventoji 2 | Lagoon | Šv2-2yA(1) | Globular Amphora | Pot | Sooted crust | -26.2 | 10.0 | 47.6 | 6.8 | 8.2 | Vilnius | 2017 | Piličiauskas et al. 2018b |
| Šventoji 2 | Lagoon | Šv2-2yA(2) | Globular Amphora | Pot | Sooted crust | -25.8 | 10.6 | 45.5 | 7.8 | 6.8 | Vilnius | 2017 | Piličiauskas et al. 2018b |
| Šventoji 3 | Lagoon | 179 | Porous Ware | Pot | Foodcrust | -28.7 | 11.6 | 37.7 | 4.8 | 9.3 | Bradford | 2014 | Piličiauskas et al. 2018b |
| Šventoji 3 | Lagoon | 114 | Porous Ware | Pot | Sooted crust | -26.2 | 11.1 | 50.7 | 7.0 | 8.5 | Bradford | 2014 | Piličiauskas et al. 2018b |
| Šventoji 3 | Lagoon | 18 (08.16) | Porous Ware | Pot | Sooted crust | -25.7 | 11.2 | 48.2 | 7.4 | 7.6 | Bradford | 2014 | Piličiauskas et al. 2018b |
| Šventoji 3 | Lagoon | 35 (08.17) | Porous Ware | Pot | Foodcrust | -25.1 | 10.5 | 47.3 | 9.0 | 6.2 | Bradford | 2014 | Piličiauskas et al. 2018b |
| Šventoji 3 | Lagoon | 246 (09.13) | Porous Ware | Pot | Foodcrust | -27.1 | 11.2 | 46.9 | 8.8 | 6.2 | Bradford | 2014 | Piličiauskas et al. 2018b |
| Šventoji 3 | Lagoon | ASAS 85 | Porous Ware | Pot | Sooted crust | -26.7 | 11.1 | 47.5 | 7.5 | 7.4 | Bradford | 2014 | Heron et al. 2015 |
| Šventoji 4 | Lagoon | 982 | Globular Amphora | Amphora | Foodcrust | -25.9 | 1.5 | 34.8 | 2.4 | 16.9 | Bradford | 2014 | Heron et al. 2015 |
| Šventoji 4 | Lagoon | Šv4-2014-1059 | Globular Amphora | Amphora | Foodcrust | -27.3 | 4.3 | 39.8 | 3.7 | 12.5 | Vilnius | 2017 | This study |
| Šventoji 4 | Lagoon | 1234 | Globular Amphora | Pot | Foodcrust | -26.9 | 7.2 | 46.6 | 7.3 | 7.4 | Bradford | 2014 | Piličiauskas et al. 2018b |
| Šventoji 4 | Lagoon | 1413(09.14) | Globular Amphora | Pot | Sooted crust | -26.2 | 7.5 | 42.4 | 5.4 | 9.2 | Bradford | 2014 | Piličiauskas et al. 2018b |
| Šventoji 4 | Lagoon | 2 | Globular Amphora | Pot | Foodcrust | -26.5 | 7.7 | 40.0 | 6.5 | 7.2 | Bradford | 2014 | Piličiauskas et al. 2018b |
| Šventoji 4 | Lagoon | Šv4-2014-1029 | Globular Amphora | Pot | Foodcrust | -27.8 | 8.3 | 53.9 | 6.8 | 9.3 | Vilnius | 2017 | This study |
| Šventoji 4 | Lagoon | 1398 | Globular Amphora | Pot | Foodcrust | -24.5 | 8.9 | 44.0 | 5.3 | 9.8 | Bradford | 2014 | Piličiauskas et al. 2018b |
| Šventoji 4 | Lagoon | Šv4-2006-S329/3 | Globular Amphora | Pot | Foodcrust | -27.9 | 9.1 | 31.9 | 5.0 | 7.4 | Vilnius | 2017 | Piličiauskas et al. 2018b |
| Šventoji 4 | Lagoon | Šv4-XVIII-31v+ž-FD | Globular Amphora | Pot | Foodcrust | -27.1 | 9.1 | 50.4 | 9.3 | 6.3 | Vilnius | 2017 | Piličiauskas et al. 2018b |
| Šventoji 4 | Lagoon | Šv4-2014-1010 | Globular Amphora | Pot | Foodcrust | -26.2 | 9.3 | 55.4 | 8.6 | 7.5 | Vilnius | 2017 | Piličiauskas et al. 2018b |
| Šventoji 4 | Lagoon | Šv4-2006-S325/1 | Globular Amphora | Amphora | Foodcrust | -25.9 | 9.7 | 50.5 | 6.7 | 8.8 | Vilnius | 2017 | Piličiauskas et al. 2018b |
| Šventoji 4 | Lagoon | Šv4-XVIII-31v+ž-ST | Globular Amphora | Pot | Sooted crust | -26.2 | 10.0 | 40.6 | 5.8 | 8.2 | Vilnius | 2017 | Piličiauskas et al. 2018b |
| Šventoji 4 | Lagoon | Šv4-2014-1058 | Globular Amphora | Amphora | Sooted crust | -25.8 | 10.3 | 40.8 | 3.7 | 12.7 | Vilnius | 2017 | Piličiauskas et al. 2018b |
| Šventoji 4 | Lagoon | Šv4-2006-EM2136:825 | Globular Amphora | Bowl | Foodcrust | -26.3 | 10.4 | 17.1 | 1.2 | 17.0 | Vilnius | 2017 | Piličiauskas et al. 2018b |
| Šventoji 4 | Lagoon | 34 | Porous Ware | Pot | Sooted crust | -26.7 | 10.2 | 45.6 | 6.1 | 8.7 | Bradford | 2014 | Piličiauskas et al. 2018b |
| Šventoji 4 | Lagoon | 1436(09.14) | Porous Ware | Pot | Foodcrust | -27.2 | 11.2 | 40.8 | 8.4 | 5.7 | Bradford | 2014 | Piličiauskas et al. 2018b |
| Šventoji 4 | Lagoon | 333 | Porous Ware | Pot | Foodcrust | -26.3 | 10.3 | 41.8 | 8.4 | 5.8 | Bradford | 2014 | Piličiauskas et al. 2018b |
| Šventoji 4 | Lagoon | 530 | Porous Ware | Pot | Foodcrust | -26.4 | 11.9 | 40.4 | 8.6 | 5.5 | Bradford | 2014 | Piličiauskas et al. 2018b |
| Šventoji 4 | Lagoon | 44/1 | Porous Ware | Pot | Foodcrust | -25.3 | 13.2 | 30.0 | 5.7 | 6.1 | Bradford | 2014 | Piličiauskas et al. 2018b |
| Šventoji 4 | Lagoon | 1677 | Porous Ware | Pot | Foodcrust | -26.7 | 10.8 | 40.1 | 7.2 | 6.5 | Bradford | 2014 | Piličiauskas et al. 2018b |
| Šventoji 4 | Lagoon | 111 | Porous Ware | Pot | Foodcrust | -26.1 | 11.4 | 37.0 | 7.8 | 5.5 | Bradford | 2014 | Piličiauskas et al. 2018b |
| Šventoji 4 | Lagoon | Prk2/115 | Porous Ware | Prolonged bowl | Foodcrust | -31.7 | 11.2 | 52.9 | 3.8 | 17.0 | Bradford | 2014 | Heron et al. 2015 |
| Šventoji 47 | Lagoon | 285 | Post-Corded Ware | Pot | Foodcrust | -25.9 | 4.5 | 42.9 | 3.5 | 14.3 | Bradford | 2014 | Piličiauskas et al. 2018b |
| Šventoji 47 | Lagoon | Šv47-2013-262 | Post-Corded Ware | Pot | Sooted crust | -26.5 | 6.9 | 48.8 | 4.3 | 13.2 | Vilnius | 2017 | Piličiauskas et al. 2018b |
| Šventoji 6 | Lagoon | Šv6-51j | Globular Amphora | Bowl | Foodcrust | -28.5 | 6.4 | 44.7 | 4.6 | 11.4 | Vilnius | 2017 | Piličiauskas et al. 2018b |
| Šventoji 6 | Lagoon | Šv6-42j | Globular Amphora | Pot | Foodcrust | -28.6 | 10.7 | 52.2 | 5.9 | 10.4 | Vilnius | 2017 | Piličiauskas et al. 2018b |
| Šventoji 6 | Lagoon | Šv6-16i | Globular Amphora | Pot | Sooted crust | -27.3 | 10.9 | 50.1 | 6.0 | 9.8 | Vilnius | 2017 | Piličiauskas et al. 2018b |
| Šventoji 6 | Lagoon | EM2138:1410 | Porous Ware | Pot | Sooted crust | -26.9 | 12.3 | 47.0 | 6.0 | 9.2 | Bradford | 2014 | Piličiauskas et al. 2018b |
| Šventoji 6 | Lagoon | 31g | Porous Ware | Prolonged bowl | Foodcrust | -29.5 | 9.9 | 119.5 | 5.0 | 28.6 | Bradford | 2014 | Heron et al. 2015 |
| Šventoji 9 | Lagoon | EM2140:6 | Post-Corded Ware | Pot | Foodcrust | -26.3 | 4.0 | 65.1 | 5.4 | 14.1 | Bradford | 2014 | Piličiauskas et al. 2018b |
| Žemaitiškė 1 | Inland | Zm1-J5 | Hybrid Ware | Pot | Foodcrust | -28.0 | 8.3 | 31.9 | 5.0 | 7.5 | Vilnius | 2017 | Piličiauskas et al. 2018b |
| Žemaitiškė 1 | Inland | Zm1-O3 | Hybrid Ware | Pot | Foodcrust | -29.1 | 7.4 | 55.4 |  |  | Vilnius | 2017 | Piličiauskas et al. 2018b |
| Žemaitiškė 1 | Inland | Zm1-P7 | Hybrid Ware | Pot | Foodcrust | -26.5 | 8.7 | 35.3 | 4.9 | 8.5 | Vilnius | 2017 | Piličiauskas et al. 2018b |
| Žemaitiškė 1 | Inland | Zm1-K4 | Hybrid Ware | Pot | Foodcrust | -29.6 | 9.0 | 38.0 | 6.1 | 7.3 | Vilnius | 2017 | Piličiauskas et al. 2018b |
| Žemaitiškė 3 | Inland | Zm3-84L37 | Hybrid Ware | Pot | Sooted crust | -28.0 | 11.8 | 51.1 | 4.7 | 12.7 | Vilnius | 2017 | Piličiauskas et al. 2018b |

***Table S4. Summary of identified compounds from the acidified methanol and solvent extracts. Struck-through, lipid concentrations that were below the minimum amount required for interpretation.***

| **Sample code** | **Acid extracts** | | | | | | | | | **Solvent extrats** | | |
| --- | --- | --- | --- | --- | --- | --- | --- | --- | --- | --- | --- | --- |
|  | **Lipid conc. (ug/g)** | **Saturated fatty acids** | | **Unsaturated fatty acids** | | **Branched fatty acids** | **Terpenes** | **Ketones** | *n*-**Alkanes** | **Acyl lipids** | **Terpenes** | **Other** |
|  |  | **Range** | **Dominant** | **Monounsaturated fatty acids** | **Polyunsaturated fatty acids** |  |  |  |  |  |  |  |
| ***Alksnynė 3*** | | | | | | | | | | | | |
| Al1 | ~~83.0~~ | C16:0-C26:0 | C16:0; C18:0 | C16:1; C18:1; C20:1; C22:1; C24:1 | C18:2 |  |  |  |  |  |  |  |
| Al2 | 173.2 | C10:0-C24:0 | C16:0; C18:0 | C16:1 |  | C15:0; C17:0 |  | 14-K29; 16-K31; 16-K33; 18-K35 |  | MAGs; DAGs |  |  |
| Al3 | 116.5 | C14:0-C24:0 | C16:0; C18:0 | C16:1; C18:1 |  | C15:0; C17:0 |  |  |  |  |  |  |
| Al4 | ~~86.8~~ | C14:0-C24:0 | C18:0; C16:0 | C16:1; C18:1; C20:1; C22:1; C24:1 |  | C15:0; C17:0 |  |  |  |  |  |  |
| Al5 | 165.2 | C11:0-C24:0 | C16:0; C18:0 | C16:1; C18:1; C22:1; C24:1 | C18:2 | C15:0; C17:0 |  |  |  |  |  |  |
| Al6 | 151.9 | C11:0-C28:0 | C16:0; C18:0 | C16:1; C18:1 |  | C15:0; C17:0 |  | 14-K29; 16-K31; 16-K33; 18-K35 |  | MAGs; DAGs; TAGs |  |  |
| Al7 | 12.0 | C14:0-C26:0 | C16:0; C18:0 | C16:1; C18:1; C20:1; C22:1; C24:1 | C18:2 | C15:0; C17:0 |  |  |  | MAGs; DAGs |  |  |
| Al8 | 17.5 | C11:0-C28:0 | C16:0; C18:0 | C16:1; C18:1; C20:1; C22:1; C24:1 |  | C15:0; C17:0 |  |  |  | MAGs; DAGs |  |  |
| Al9 | 10.3 | C10:0-C24:0 | C16:0; C18:0 | C16:1; C18:1; C20:1; C22:1; C24:1 | C18:2 |  |  |  |  |  |  |  |
| Al10 | 5.4 | C14:0-C24:0 | C16:0; C18:0 | C18:1; C20:1; C22:1; C24:1 | C18:2 | C15:0; C17:0 |  |  |  |  |  |  |
| ***Daktariškė 5*** | | | | | | | | | | | | |
| DK V 4a2 | 3818.2 | C14:0-C24:0 | C16:0; C18:0 | C16:1; C18:1; C20:1 | C18:2 | C15:0; C17:0 |  |  |  |  |  |  |
| Dk V 8a | 67.4 | C12:0-C26:0 | C16:0; C18:0 | C16:1; C18:1; C20:1; C22:1 |  | C15:0 | Betulin | 16-K31 |  |  | Present | ALK (20-31) odd chain pred. C29/LC alkanols (C24-C26) |
| Dk X 8b1 | 6245.6 | C13:0-C20:0 | C16:0; C18:0 | C16:1; C18:1 |  | C15:0; C17:0 |  |  |  |  |  |  |
| Dk108 | 24.1 | C12:0-C26:0 | C16:0; C18:0 | C16:1; C18:1 |  | C15:0; C17:0 | Betulin |  |  |  | Unidentified terpenes |  |
| Dk240 | 2602.3 | C13:0-C28:0 | C16:0; C18:0 | C16:1; C18:1; C20:1 |  | C15:0; C17:0 |  |  |  |  | Unidentified terpenes |  |
| Dk323 | 791.7 | C14:0-C28:0 | C16:0; C18:0 | C16:1; C18:1; C20:1; C22:1 |  | C15:0; C17:0 | Betulin |  |  |  | Unidentified terpenes |  |
| DK342 | 10923.1 | C14:0-C28:0 | C16:0; C18:0 | C16:1; C18:1; C20:1 | C18:2 | C15:0; C17:0 | β-Amyrin |  |  |  |  |  |
| Dk417 | 3708.7 | C6:0-C24:0 | C16:0; C18:0 | C16:1; C18:1 |  | C15:0 |  |  |  |  |  |  |
| Dk419 | 6.5 | C14:0-C28:0 | C16:0; C18:0 | C16:1; C18:1; C20:1; C22:1 |  | C15:0 |  |  |  |  |  |  |
| Dk466 | 143.9 | C10:0-C26:0 | C16:0; C14:0 | C16:1; C18:1 |  | C15:0; C17:0 | Betulin |  |  | MAGs; DAGs; TAGs | Amyrin and unidentified terpenes |  |
| Dk482 | 4.7 | C14:0-C28:0 | C18:0; C16:0 | C16:1; C18:1; C20:1; C22:1 |  | C15:0; C17:0 |  |  |  |  |  |  |
| Dk492 | 866.6 | C14:0-C28:0 | C16:0; C18:0 | C16:1; C18:1; C20:1; C22:1; C24:1 |  | C15:0; C17:0 | α-Amyrin; β-Amyrin; Betulin; Lupeol |  | Main C29 trace |  | Amyrin and unidentified terpenes |  |
| DK494 | 2180.6 | C14:0-C28:0 | C16:0; C18:0 | C16:1; C18:1; C20:1; C24:1 |  | C15:0; C17:0 | β-Amyrin; Betulin; Lupeol |  |  |  |  |  |
| Dk524 | 1632.3 | C14:0-C26:0 | C16:0; C18:0 | C16:1; C18:1; C20:1 |  | C15:0; C17:0 | Betulin; Lupeol |  |  |  | Unidentified terpenes |  |
| Dk525 | 598.1 | C14:0-C26:0 | C16:0; C18:0 | C16:1; C18:1; C20:1; C22:1; C24:1 |  | C15:0; C17:0 |  |  | Main C29 trace |  |  |  |
| Dk541 | 920.6 | C14:0-C28:0 | C16:0; C18:0 | C16:1; C18:1 |  | C15:0; C17:0 |  |  | Main C29 trace |  |  |  |
| Dk623 | 214.4 | C14:0-C30:0 | C18:0; C16:0 | C16:1; C18:1; C20:1; C22:1 |  | C15:0; C17:0 |  |  | Main C29 trace |  |  |  |
| DK87 I 4a | 5927.6 | C7:0-C24:0 | C16:0; C18:0 | C16:1; C18:1 | C18:2 | C15:0; C17:0 | Betulin; Lupeol |  |  |  |  |  |
| DK87 I kontr. 1 | 261369.0 | C12:0-C24:0 | C18:0; C16:0 | C16:1; C18:1; C22:1; C24:1 | C18:2 | C15:0; C17:0 | α-Amyrin |  |  |  |  |  |
| DK87 II 6a | 46140.1 | C14:0-C24:0 | C16:0; C18:0 | C16:1; C18:1 | C18:2 | C15:0; C17:0 | α-Amyrin; β-Amyrin; Betulin; Lupeol |  |  |  | Amyrin |  |
| DK87 III 3a1 | 10560.0 | C14:0-C26:0 | C16:0; C18:0 | C16:1; C18:1 | C22:5 | C17:0 | β-Amyrin; Betulin |  |  |  | Amyrin |  |
| DK87 ST | 2246.3 | C13:0-C28:0 | C16:0; C18:0 | C16:1; C18:1; C20:1; C24:1 |  | C15:0; C17:0 |  |  |  |  |  |  |
| DK87 V 4b | 57265.3 | C13:0-C24:0 | C18:0; C16:0 | C16:1; C18:1; C20:1 | C18:2 | C15:0; C17:0 |  |  |  |  |  |  |
| DK87 V 6a3 | 2984.1 | C14:0-C22:0 | C16:0; C18:0 | C16:1; C18:1; C24:1 | C18:2 | C15:0; C17:0 | α-Amyrin; β-Amyrin; Betulin |  |  |  |  |  |
| DK88 VII 5b | 14752.0 | C13:0-C28:0 | C18:0; C16:0 | C16:1; C18:1 |  | C15:0; C17:0 | Lupeol |  |  |  |  |  |
| DK88 VIII 6a1 | 2127.8 | C13:0-C26:0 | C16:0; C18:0 | C18:1 |  | C17:0 |  |  |  |  |  |  |
| DK90 X 6b2 | 692.4 | C4:0-C28:0 | C16:0; C18:0 |  |  | C17:0 |  |  |  |  |  |  |
| ***Dubičiai 2*** | | | | | | | | | | | | |
| Db2 perk. 7 | 8.0 | C7:0-C18:0 | C16:0; C18:0 | C16:1; C18:1 |  | C17:0 |  |  | Present |  |  |  |
| Db2 perk. 8 | 10.0 | C7:0-C18:0 | C16:0; C14:0 | C16:1; C18:1 |  | C15:0; C17:0 |  |  | Present |  |  |  |
| ***Gribaša 4*** | | | | | | | | | | | | |
| vessel No 3 | 8.8 | C11:0-C24:0 | C16:0; C18:0 | C16:1; C18:1 |  | C15:0; C17:0 |  |  |  |  |  |  |
| vessel No 20 | 12.5 | C12:0-C18:0 | C16:0; C18:0 | C16:1; C18:1; C22:1 |  | C15:0; C17:0 |  |  |  |  |  |  |
| ***Karaviškės 6*** | | | | | | | | | | | | |
| H-32 | 82.1 | C14:0-C20:0 | C18:0; C16:0 | C16:1; C18:1 |  | C15:0; C17:0 |  |  | Present |  |  |  |
| EM2502:523 | 5.6 | C12:0-C18:0 | C14:0; C16:0 | C16:1; C18:1 |  | C15:0; C17:0 |  |  | Present |  |  |  |
| EM2502:533 | ~~4.3~~ | C16:0-C22:0 | C18:0; C16:0 | C16:1 trace; C18:1; C22:1 |  | C17:0 |  |  | Present |  |  |  |
| EM2502:560 | 32.2 | C9:0-C20:0 | C14:0; C16:0 | C18:1 |  |  |  |  |  |  |  |  |
| ***Kvietiniai*** | | | | | | | | | | | | |
| Kv1035 | 432.7 | C14:0-C24:0 | C18:0; C16:0 | C16:1; C18:1 |  | C15:0; C17:0 |  | 14-K29; 16-K31; 16-K33; 18-K35 |  |  |  |  |
| Kv1298 | 268.7 | C6:0-C28:0 | C16:0; C18:0 | C16:1; C18:1; C20:1; C22:1; C24:1 |  | C15:0; C17:0 |  | 14-K29; 16-K31; 16-K33; 18-K35 |  | MAGs; DAGs; TAGs |  |  |
| Kv152 | 45.4 | C12:0-C26:0 | C16:0; C18:0 | C16:1; C18:1; C20:1; C22:1; C24:1 |  | C15:0; C17:0 |  |  |  | MAGs; DAGs; TAGs |  |  |
| Kv1564 | 384.7 | C14:0-C26:0 | C18:0; C16:0 | C16:1; C18:1 |  | C15:0; C17:0 |  |  |  |  |  |  |
| Kv1588 | 68.1 | C11:0-C24:0 | C18:0; C16:0 | C18:1 |  | C15:0; C17:0 |  | 14-K29; 16-K31; 16-K33; 18-K35 |  |  |  |  |
| Kv2058 | 18.6 | C12:0-C26:0 | C18:0; C16:0 | C16:1; C18:1; C20:1; C22:1 |  | C15:0; C17:0 |  |  |  |  |  |  |
| Kv228 | 493.2 | C14:0-C28:0 | C18:0; C16:0 | C16:1; C18:1 |  | C15:0; C17:0 |  |  |  |  |  |  |
| Kv2303 | 13.9 | C11:0-C26:0 | C16:0; C18:0 | C16:1; C18:1; C20:1; C22:1 |  | C15:0 |  |  |  |  |  |  |
| Kv732 | ~~20.3~~ | C16:0-C28:0 | C18:0; C16:0 | C18:1; C22:1 |  |  |  |  |  |  |  |  |
| Kv1349 | 28.6 | C14:0-C22:0 | C18:0; C16:0 | C16:1; C18:1 |  | C15:0; C17:0 |  |  |  |  |  |  |
| Kv1895 | ~~3.5~~ | C10:0-C18:0 | C18:0; C16:0 | C18:1 |  | C15:0 |  |  |  |  |  |  |
| Kv1878 | 271.3 | C16:0-C24:0 | C18:0; C16:0 | C16:1; C18:1; C22:1 trace |  | C15:0; C17:0 |  |  |  |  |  |  |
| ***Neravai*** | | | | | | | | | | | | |
| Nr 14(38) | 5.4 | C11:0-C22:0 | C16:0; C18:0 | C16:1; C18:1 |  | C15:0; C17:0 |  |  | Present |  |  |  |
| ***Nida*** | | | | | | | | | | | | |
| N78_3/4c | 12.5 | C10:0-C22:0 | C16:0; C18:0 | C14:1 trace; C16:1; C18:1 |  | C15:0; C17:0 |  |  |  |  |  |  |
| N75_I/9av | 83.6 | C14:0-C18:0 | C16:0; C18:0 | C14:1; C16:1; C18:1; C20:1; C22:1 trace |  | C15:0; C17:0 |  |  |  |  |  |  |
| N75_I/22b | 16.6 | C14:0-C18:0 | C14:0; C16:0 | C14:1; C16:1; C18:1; C20:1 trace |  | C15:0; C17:0 |  |  |  |  |  |  |
| ***Šventoji 1*** | | | | | | | | | | | | |
| Šv1-B14b | 1364.6 | C14:0-C26:0 | C16:0; C18:0 | C16:1; C18:1; C20:1; C22:1; C24:1 |  | C15:0; C17:0 | Betulin; Lupeol |  | Main C29 trace |  |  | Complex mixture of terpenes |
| ***Šventoji 4*** | | | | | | | | | | | | |
| Šv4-2014-1029 | 2950.1 | C5:0-C28:0 | C16:0; C18:0 | C16:1; C18:1; C22:1; C24:1 |  | C15:0; C17:0 |  |  |  |  |  |  |
| Šv4-2014-1059 | 596.5 | C13:0-C28:0 | C16:0; C18:0 | C16:1; C18:1 |  | C15:0; C17:0 | α-Amyrin |  | Main C29 trace |  |  |  |

***Table S5. Summary of the isoprenoid fatty acids and ω-(o-alkylphenyl)alkanoic acids (APAAs) identified. TMTD - 4,8,12-trimethyltridecanoic acid, SRR% - ((Area SSR/Area SSR+Area RRR)*100).***

| **Ware** | **Vessel type** | **Sample code** | **TMTD** | **Pristanic acid** | **%SRR** | **APAAs** |
| --- | --- | --- | --- | --- | --- | --- |
| ***Alksnynė 3*** | | | | | | |
| Corded | Pot | AL01 |  |  | 71.5 |  |
| Corded | Pot | AL02 | yes | yes | 33.5 | C16; C18; C20; C22 |
| Corded | Pot | AL03 |  | yes | 36 | C16; C18; C20 |
| Corded | Pot | AL04 |  | yes | 21.8 | C16; C18; C20 |
| Corded | Pot | AL05 |  | yes | 24.7 | C16; C18; C20; C22 trace |
| Corded | Pot | AL06 | yes | yes | 40.8 | C16; C18; C20; C22 trace |
| Corded | Pot | AL07 |  | yes | 45.2 | C18 trace |
| Corded | Beaker | AL08 |  | yes | 21.5 | C16 trace; C18 trace |
| Corded | Pot | AL09 |  | yes | 38.8 |  |
| Corded | Pot | AL10 |  | yes | 25.4 |  |
| ***Daktariškė 5*** | | | | | | |
| Globular Amphora | Wide-mouth pot | DK X 6b2 |  | yes | 35.7 | C18 |
| Globular Amphora | Pot | DK X 8b1 | yes | yes | 35.2 | C16; C18; C20 |
| Post-Corded Ware | Pot | DK108 |  | yes | 59.4 | C18 |
| Corded | Beaker | DK240 |  | yes | 61.7 | C16; C18; C20 trace |
| Corded | Pot | DK323 |  | yes | 15.1 | C16; C18; C20 |
| Post-Corded Ware | Pot | DK342 |  | yes | 35.2 | C16; C18; C20 trace |
| Post-Corded Ware | Pot | DK417 | yes | yes | 30.8 | C16; C18; C20 trace |
| Post-Corded Ware | Beaker | DK419 |  | yes | 62.6 |  |
| Corded | Beaker | DK466 |  | yes | 63.8 | C16; C18; C20 |
| Post-Corded Ware | Pot | DK482 |  | yes | 64.4 |  |
| Post-Corded Ware | Pot | DK492 |  | yes | 28.3 | C16; C18; C20 |
| Post-Corded Ware | Pot | DK494 |  | yes | 32 | C16; C18; C20 trace |
| Post-Corded Ware | Pot | DK524 | yes | yes | 27.7 | C16; C18; C20; C22 trace |
| Corded | Pot | DK525 |  | yes | 29.4 | C16; C18 |
| Corded | Pot | DK541 |  | yes | 45 | C16; C18; C20 |
| Post-Corded Ware | Pot | DK623 |  | yes | 43.6 | C16; C18; C20 |
| Hybrid | Pot | DK87 I4a |  | yes | 36.9 | C18 |
| Corded | Pot | DK87 II6a |  | yes | 40.3 | C16; C18; C20 trace |
| Corded | Amphora | DK87 III 3al | yes | yes | 46.2 | C16; C18; C20 trace |
| Globular Amphora | Pot | DK87 ST |  | yes | 34 | C16; C18; C20; C22 trace |
| Corded | Beaker | DK87 V 4b |  | yes | 53.9 | C16; C18; C20 |
| Hybrid | Pot | DK87 V 6a3 |  | yes | 15.8 | C16; C18; C20 |
| Globular Amphora | Pot | DK87 I kontr. 1 |  |  |  | C16; C18; C20 trace |
| Corded | Pot | DK88 VII 5b | yes | yes | 39.3 | C16; C18; C20 |
| Hybrid | Pot | DK88 VIII 6al | yes | yes | 25.2 | C16; C18; C20 trace |
| Corded | Pot | DK V 4a2 | yes | yes | 44.8 | C16; C18; C20 |
| Globular Amphora | Bowl | DK V 8a | yes | yes | 56.5 |  |
| ***Dubičiai 2*** | | | | | | |
| Corded | Beaker | Db2 perk. 7 |  |  |  | C16 trace; C18 trace |
| Corded | Beaker | Db2 perk. 8 |  |  |  | C16 trace |
| ***Gribaša 4*** | | | | | | |
| Globular Amphora | Amphora | vessel No 3 |  |  |  |  |
| Globular Amphora | Pot | vessel No 20 |  |  |  |  |
| ***Karaviškės 6*** | | | | | | |
| Corded | Beaker | H-32 |  |  | present | C18 |
| Corded | Amphora | EM2502:523 |  |  |  |  |
| Corded | Beaker | EM2502:533 |  |  | trace | C16 trace |
| Corded | Beaker | EM2502:560 |  |  |  |  |
| ***Kvietiniai*** | | | | | | |
| Post-Corded Ware | Pot | KV1035 |  | yes | 32.2 | C16; C18; C20 trace |
| Post-Corded Ware | Pot | KV1298 |  |  | 73 | C16 trace; C18 trace |
| Corded | Beaker | KV152 |  | yes | 30 | C18 trace |
| Post-Corded Ware | Pot | KV1564 |  | yes | 51.2 | C16; C18; C20 |
| Post-Corded Ware | Pot | KV1588 |  | yes | 37.1 | C16; C18; C20 |
| Corded | Beaker | KV2058 |  | yes | 52 | C18 trace |
| Post-Corded Ware | Pot | KV228 |  | yes | 38.2 | C16; C18; C20 trace |
| Corded | Beaker | KV2303 |  | yes | 55.3 | C18 |
| Post-Corded Ware | Pot | KV732 |  |  | 61.5 |  |
| Corded | Beaker | Kv1349 |  |  | trace | C16 trace; C18 trace |
| Corded | Beaker | Kv1895 |  |  |  |  |
| Corded | Beaker | Kv1878 |  |  | trace | C16; C18 trace |
| ***Neravai*** | | | | | | |
| Corded | Beaker | Nr 14(38) |  |  |  | C16 |
| ***Nida*** | | | | | | |
| Rzucewo | Beaker | N78_3/4c | trace |  |  | C16; C18 trace |
| Rzucewo | Beaker | N75_I/9av |  |  | present | C16 trace |
| Rzucewo | Beaker | N75_I/22b |  |  |  |  |
| ***Šventoji 1*** | | | | | | |
| Corded | Pot | SV1-B-14b | yes | yes | 69.7 | C18; C20 |
| ***Šventoji 4*** | | | | | | |
| Globular Amphora | Amphora | SV4-2014-1029 | yes | yes | 23.6 | C18; C20 |
| Globular Amphora | Pot | SV4-2014-1059 |  | yes | 63 | C18; C20 trace |

***Table S6. Carbon stable isotope values of* n*-hexadecanoic (C_16:0_) and* n*-octadecanoic (C_18:0_) acid methyl esters obtained for 48/64 samples analysed in this study.***

| **Sample code** | **δ^13^C_16:0_ (**‰) | **δ^13^C_18:0_ (**‰) | **Δ^13^C (δ^13^C_18:0_-δ^13^C_16:0_)** |
| --- | --- | --- | --- |
| ***Alksnynė 3*** | | | |
| Al02 | -28.4 | -30.1 | -1.7 |
| Al06 | -29.7 | -30.9 | -1.2 |
| Al07 | -29.0 | -29.7 | -0.7 |
| Al08 | -28.3 | -29.8 | -1.5 |
| ***Daktariškė 5*** | | | |
| DK V 4a2 | -30.3 | -31.9 | -1.7 |
| DK V 8a | -30.8 | -30.3 | 0.5 |
| Dk X 8b1 | -30.9 | -32.1 | -1.2 |
| DK240 | -28.1 | -32.4 | -4.3 |
| DK323 | -31.4 | -30.6 | 0.8 |
| DK342 | -32.0 | -31.6 | 0.4 |
| DK417 | -30.8 | -30.2 | 0.5 |
| DK466 | -29.3 | -33.7 | -4.4 |
| DK492 | -33.2 | -32.5 | 0.7 |
| DK494 | -29.2 | -29.5 | -0.3 |
| DK524 | -29.9 | -30.1 | -0.2 |
| DK525 | -33.0 | -32.6 | 0.3 |
| DK541 | -30.8 | -32.0 | -1.2 |
| DK623 | -29.7 | -34.4 | -4.7 |
| DK87 I 4a | -29.7 | -30.8 | -1.1 |
| DK87 I kontr. 1 | -29.6 | -34.5 | -4.9 |
| DK87 II 6a | -33.9 | -32.3 | 1.6 |
| DK87 III 3a1 | -34.7 | -34.1 | 0.5 |
| DK87 ST | -31.0 | -31.9 | -0.9 |
| DK87 V 4b | -28.8 | -31.7 | -2.8 |
| DK87 V 6a3 | -31.2 | -30.5 | 0.7 |
| DK88 VII 5b | -28.0 | -29.6 | -1.6 |
| DK88 VIII 6a1 | -31.8 | -30.8 | 1.0 |
| ***Dubičiai 2*** | | | |
| Db2 perk. 7 | -28.9 | -29.3 | -0.4 |
| Db2 perk. 8 | -27.4 | -28.5 | -1.1 |
| ***Gribaša 4*** | | | |
| vessel No 3 | -28.3 | -29.3 | -1.1 |
| ***Karaviškės 6*** | | | |
| H-32 | -31.5 | -32.8 | -1.3 |
| EM2502:533 | -30.0 | -30.1 | -0.1 |
| EM2502:560 | -28.3 | -29.9 | -1.6 |
| ***Kvietiniai*** | | | |
| KV1035 | -27.4 | -32.8 | -5.4 |
| KV1298 | -27.2 | -27.6 | -0.4 |
| KV152 | -29.3 | -35.8 | -6.5 |
| KV1564 | -28.8 | -33.5 | -4.7 |
| KV1588 | -27.9 | -29.2 | -1.3 |
| KV2058 | -29.0 | -31.9 | -2.9 |
| KV228 | -27.9 | -30.4 | -2.5 |
| KV2303 | -28.9 | -30.4 | -1.6 |
| Kv1349 | -30.3 | -31.7 | -1.4 |
| Kv1895 | -30.5 | -29.4 | 1.1 |
| Kv1878 | -29.3 | -32.5 | -3.2 |
| ***Nida*** | | | |
| N75_I/9av | -31.5 | -31.4 | 0.1 |
| N75_I/22b | -30.5 | -33.5 | -2.9 |
| ***Šventoji 1*** | | | |
| Šv1-B14b | -28.6 | -30.6 | -2.0 |
| ***Šventoji 4*** | | | |
| Šv4-2014-1059 | -30.5 | -31.1 | -0.7 |

***Table S7. Carbon stable isotope values of n-hexadecanoic (C_16:0_) and n-octadecanoic (C_18:0_) acid methyl esters obtained from contemporaneous vessels throughout the circum-Baltic region that are discussed in the article.***

| **Site name** | **Country** | **Ware** | **Sample code** | **Vessel type** | **δ^13^C_16:0_ (‰)** | **δ^13^C_18:0_ (‰)** | **Δ^13^C (‰)** | **Reference** |
| --- | --- | --- | --- | --- | --- | --- | --- | --- |
| Dakudava 5 | Belarus | Corded Ware | Dakudava5 | Beaker | -29.3 | -34.4 | -5.1 | Piličiauskas et al. 2018a |
| Drazdy 12 | Belarus | Corded Ware | Drazdy12 | Beaker | -28.4 | -30.0 | -1.7 | Piličiauskas et al. 2018a |
| Kirkkonummi Backisåker I (Kvarnåker) | Finland | Corded Ware | KM-45i 7349 : 5 | Large 'S-shaped' amphora | -26.5 | -29.8 | -3.3 | Cramp et al. 2014a |
| Kirkkonummi Backisåker I (Kvarnåker) | Finland | Corded Ware | KM-57 5944 : 46 | Decorated beaker | -27.7 | -31.8 | -4.1 | Cramp et al. 2014a |
| Kirkkonummi Koivistosveden | Finland | Corded Ware | KM-44i; 7734 : 11 | Beaker | -24.2 | -23.8 | 0.4 | Cramp et al. 2014a |
| Kirkkonummi Tengå Nyåker | Finland | Corded Ware | KM-1; 8709 : 52 | Beaker | -28.6 | -34.3 | -5.7 | Cramp et al. 2014a |
| Kirkkonummi Tengå Nyåker | Finland | Corded Ware | KM-31; 8709 : 35 | S-shaped' amphora | -27.1 | -29.8 | -2.7 | Cramp et al. 2014a |
| Kirkkonummi Tengå Nyåker | Finland | Corded Ware | KM-47; 8709 : 17 | Large beaker, impressed decoration | -27.7 | -30.0 | -2.3 | Cramp et al. 2014a |
| Kirkkonummi Tengå Nyåker | Finland | Corded Ware | KM-48; 8709 : 22 | Large beaker | -25.8 | -31.4 | -5.6 | Cramp et al. 2014a |
| Nida 1 | Lithuania | Rzucewo | L2i | Beaker | -32.0 | -32.5 | -0.5 | Heron et al. 2015 |
| Nida 1 | Lithuania | Rzucewo | L3i | Beaker | -28.5 | -32.6 | -4.1 | Heron et al. 2015 |
| Nida 1 | Lithuania | Rzucewo | L4i | Wide-mouthed pot | -31.4 | -31.3 | 0.1 | Heron et al. 2015 |
| Nida 1 | Lithuania | Rzucewo | L5i | Pot | -31.2 | -31.3 | -0.1 | Heron et al. 2015 |
| Nida 1 | Lithuania | Rzucewo | L6i | Wide-mouthed pot | -30.9 | -31.7 | -0.8 | Heron et al. 2015 |
| Nida 1 | Lithuania | Rzucewo | L7i | Pot | -30.1 | -30.8 | -0.7 | Heron et al. 2015 |
| Nida 1 | Lithuania | Rzucewo | L8iA | Large pot | -32.2 | -32.6 | -0.4 | Heron et al. 2015 |
| Nida 1 | Lithuania | Rzucewo | L8iB | Large pot | -31.1 | -31.3 | -0.2 | Heron et al. 2015 |
| Nida 1 | Lithuania | Rzucewo | L9i | Pot | -31.9 | -32.6 | -0.7 | Heron et al. 2015 |
| Nida 1 | Lithuania | Rzucewo | L10i | Wide-mouthed pot | -29.9 | -30.6 | -0.7 | Heron et al. 2015 |
| Nida 1 | Lithuania | Rzucewo | L11i | Wide-mouthed pot | -25.8 | -28.3 | -2.5 | Heron et al. 2015 |
| Nida 1 | Lithuania | Rzucewo | L12i | Prolonged bowl/lamp | -32.2 | -32.3 | -0.1 | Heron et al. 2015 |
| Nida 1 | Lithuania | Rzucewo | L16i | Wide-mouthed pot | -32.0 | -32.5 | -0.5 | Heron et al. 2015 |
| Nida 1 | Lithuania | Rzucewo | L19i | Beaker | -30.7 | -32.2 | -1.5 | Heron et al. 2015 |
| Nida 1 | Lithuania | Rzucewo | L21i | Beaker | -28.0 | -33.4 | -5.4 | Heron et al. 2015 |
| Nida 1 | Lithuania | Rzucewo | L25i | Prolonged bowl/lamp | -32.1 | -33.1 | -1.0 | Heron et al. 2015 |
| Šventoji 4 | Lithuania | Globular Amphora ware | L1i | Amphora | -29.1 | -28.8 | 0.3 | Heron et al. 2015 |
| Šventoji 4 | Lithuania | Globular Amphora ware | L17i | Amphora | -29.7 | -29.8 | -0.1 | Heron et al. 2015 |
