# Supplementary material for: Diet, cuisine and consumption practices of the first farmers in the southeastern Baltic
Source: Archaeol Anthropol Sci. 2019 Feb 15;11(8):4011–24. doi: 10.1007/s12520-019-00804-9 (PMC6743674; doi:10.1007/s12520-019-00804-9)
Supplement: Supplementary file 2 — (DOCX 38.2 kb) [file 12520_2019_804_MOESM2_ESM.docx]

**Supplementary information for Diet, cuisine and consumption practices of the first farmers in the south-eastern Baltic**

Harry K. Robson^1*^, Raminta Skipitytė^2, 5^, Giedrė Piličiauskienė^3^, Alexandre Lucquin^1^, Carl Heron^4^, Oliver E. Craig^1^ and Gytis Piličiauskas^5*^

¹BioArCh, Department of Archaeology, University of York, Heslington, York YO10 5DD, UK

^2^Center for Physical Sciences and Technology, Saulėtekio ave. 3, Vilnius 10257, Lithuania

^3^Faculty of History, Vilnius University, Universiteto st. 7, Vilnius 01513, Lithuania

^4^Department of Scientific Research, The British Museum, Great Russell Street, London WC1B 3DG, UK

^5^Lithuanian Institute of History, Kražių st. 5, Vilnius 01108, Lithuania

*corresponding authors: hkrobson@hotmail.co.uk and gytis.piliciauskas@gmail.com

**This word document includes:**

Supplementary Text

References for Supplementary Text and Tables S1 to S7

**Supplementary Text**

**S1. Background information pertaining to the sampled sites**

All of the sites sampled in this study are located in Lithuania in the south-eastern Baltic. Four of them are situated on the coast (Alksnynė 3, Nida, Šventoji 1 and Šventoji 4), whilst six are inland localities (Daktariškė 5, Dubičiai 2, Gribaša 4, Karaviškės 6, Kvietiniai and Neravai).

**Coastal sites**

Alksnynė 3 is situated on the Curonian Spit, a narrow band of land separating the Baltic Sea from the Curonian Lagoon, in south-western Lithuania. Alksnynė 3 has been dated to the Corded Ware culture (CWC), *ca*. 2600/2400 cal BC. The faunal assemblage was composed of 61 specimens, which were identified to the following species: cattle (*Bos taurus*), pig (*Sus* sp.), sheep/goat (Ovicaprid), unidentified seal (Phocidae), roe deer (*Capreolus capreolus*), northern pike (*Esox lucius*), pikeperch (*Zander lucioperca*) and common bream (*Abramis brama*). The assemblage indicates a mixed economy, including animal husbandry, freshwater fishing from the Curonian Lagoon, and seal hunting. Ten CWC vessels from Alksnynė 3 were selected for analysis.

The site of Nida is similarly located on the Curonian Spit. An area of *ca*. 4640 m^2^ was uncovered by Rimantienė (1989), and then *ca*. 160 m^2^ was excavated by one of us (Piličiauskas 2016; Piličiauskas et al. 2017a). The majority of the anthropogenic materials belonged to the Rzucewo culture (RC) although some Subneolithic pottery from previous episodes of occupation, and some probable imported CWC vessels were recovered. The RC materials have been dated from *ca*. 3200-2500 cal BC (Piličiauskas and Heron 2015). Although the zooarchaeological data demonstrates a mixed economy, there was a focus on fishing. Despite the small size of the mammalian bone assemblage, a range of species were identified, including cattle (*Bos taurus*), sheep/goat (Ovis/Aries), unidentified seal (Phocidae), beaver (*Castor fiber*), red deer (*Cervus elaphus*), elk (*Alces alces*), auroch/bison (*Bos* sp.), boar (*Sus scrofa*), fox (*Vulpes vulpes*) and horse (*Equus* sp.) (Piličiauskas 2018). Equally, the identified fish remains (NISP = *ca*. 500) included freshwater taxa (common bream (*Abramis brama*), perch (*Perca fluviatilis*), northern pike (*Esox lucius*) and pikeperch (*Zander lucioperca*)) that had probably been caught alongside anadromous fish (European eel (*Anguilla anguilla*) and Salmonidae) in the Curonian lagoon located nearby. Interestingly, vertebrae from small sized fish (<15 cm in total length) were recovered that were identified as Baltic herring (*Clupea harengus*) and sprat (*Sprattus sprattus*) (Piličiauskienė unpublished data; Schmölcke unpublished data). In total, 28 RC vessels were previously investigated by Heron et al. (2015). In this study an additional three beakers were sampled.

Šventoji 1 is located in north-western Lithuania. Here, numerous Subneolithic and Neolithic artefacts have been recovered, including a wooden fishing structure embedded into waterlogged and well-stratified gyttja. The site was excavated between 1967 and 1969 by Rimantienė (2005). Initially, all Neolithic pottery was attributed to the CWC or RC, which at the time were considered to be the same culture (Rimantienė 2005), however, during the re-analysis of the assemblage by one of us (G.P.), it is apparent that two pottery types are present (CWC, *n* = 33 vessels; Globular Amphora ware (GAC), *n* = 55 vessels), whilst RC ceramics are entirely absent. According to an age-depth model compiled for the nearby Šventoji 4 site, Subneolithic wares were replaced by GAC ware *ca*. 2700 cal BC in the area, whilst the CWC replaced the GAC perhaps over the course of several decades (Piličiauskas 2016). Since much of the pottery sherds from Šventoji 1 were heavily contaminated with conservation materials, only a single base sherd of a CWC vessel was selected for analyses.

Šventoji 4 is another wetland site that is situated in the same lagoonal lake as Šventoji 1. The main difference between the two sites is that Šventoji 4 did not yield any materials dating to the CWC. Here, the assemblage was composed of materials dating to the Late Subneolithic (*ca*. 3000-2700 cal BC) and GAC (*ca*. 2700 cal BC) (Piličiauskas 2016; Rimantienė 2005). In this study two GAC vessels were sampled.

**Inland sites**

Daktariškė 5 is located in north-western Lithuania. Here, a refuse layer within lake sediments, sandy peat and gyttja were identified. Unfortunately ploughing had destroyed the dwelling zone. Between 1987 and 1990, an area of 648 m² was investigated by Butrimas (1992). In 2016, a trench measuring 49 m^2^ was excavated by one of us (G.P.). In total, 27 sherds (CWC, *n* = 10; Post-Corded, *n* = 9; GAC, *n* = 5) from both excavation campaigns were sampled. In addition, three vessels classified as ‘Hybrid’ ware were included. These vessels had shell temper associated with the Subneolithic as well as cord ornamentation affiliated with the Neolithic.

Dubičiai 2, Gribaša 4, and Karaviškės 6 all are multi-period and unstratified sites in south-eastern Lithuania. They are all situated on the former shoreline of Lakes Duba and Pelesa (Grinevičiūtė 2002; Piličiauskas 2004; Rimantienė 1999), and have yielded CWC pottery. The site of Karaviškės 6 is presently the largest CWC site in the region and has yielded at least 37 vessels. In this study, we sampled three CWC beakers and a single amphora from Karaviškės 6. In addition, two CWC sherds from Dubičiai 2 were sampled, which may derive from the same beaker. At Gribaša 4, a small pit containing two GAC vessels - a medium size pot and an amphora - was uncovered (Grinevičiūtė 2002, Figs. 22 & 23), both of which were sampled.

Kvietiniai is a dry land site situated on a terrace of the Minija River in north-western Lithuania. In 2015, it was investigated prior to the installation of a pipeline. Here, CWC, Post-Corded and Iron Age artefacts were found within the same 0.15-0.30 m thick layer of grey and/or black fine sand (Vengalis et al. 2016). A total of 12 vessels were selected for analyses (CWC, *n* = 6; Post-Corded ware (Neolithic II-Early Bronze Age (EBA)), *n* = 6).

The site of Neravai, in south-eastern Lithuania, is renowned for numerous Iron Age burial mounds that were excavated between 1972-1977 (Kuncienė 1978). Recently, however, CWC sherds were identified among the cremated human remains from burial No. 3. The sherds belonged to two beakers. Since the location of the site, positioned on a high terrace of the Neris River, is unusual for the CWC, it is possible that the beakers originate from a burial located nearby rather than the dwelling area. One of these beakers was sampled in this study.

**S2. Instrumentation**

**Gas Chromatography-Mass Spectrometry**

Analysis by GC-MS was undertaken on an Agilent 7690A Series Gas Chromatograph coupled to an Agilent 5975C Inert XL Mass-Selective Detector with a Quadrupole Mass Analyser and Triple-Axis Detector (Agilent Technologies, Cheadle, Cheshire, UK). The splitless injector and interface were maintained at 300 °C and 340 °C respectively. The carrier gas was helium. The GC column was inserted directly into the ion source of the mass spectrometer. The ionisation energy was 70 eV and spectra were obtained by scanning between *m/z* 50 and 800. Both samples were analysed using a DB-5-ms (5%-phenyl)-methylpolysiloxane column (30 m x 0.32 mm x 0.25 μm; J&W Scientific, Folsom, CA, USA). The temperature program was set at 50 °C for 2 min, which increased to 325 °C (10 °C min^–1^). This temperature (325 °C) was held for 15 min. The data obtained via this method was used to calculate the lipid yields and identify the main molecular components. The quantification and identification of compounds was conducted with the Agilent ChemStation software according to the mass spectrum, retention time and with reference to the NIST 2008 library of mass spectra. For quantification purposes, peak integration was carried out using Agilent MSD ChemStation. Lipid concentrations, omitting contamination (for example sulphur and plasticisers), were quantified using the following formula:

[(Area (Sample)/(Area Internal Standard)) * Mass(Internal Standard)]/Mass (Sample)

The majority of the sample were then analysed using a DB-23, (50%-Cyanopropyl)-methylpolysiloxane column (60 m x 0.25 mm x 0.25 μm; J&W Scientific, Folsom, CA, USA) column in Single Ion Monitoring mode (SIM). The temperature program was set at 50 ºC for 2 min, which increased by 10 ºC per min until 100 ºC was obtained. The temperature then increased by 4 ºC per min to 140 ºC, 0.5 ºC per min to 160 ºC and 20 ºC per min to 250 ºC. This temperature (250 ºC) was held for 10 min. The first group of ions (*m/z* 74, 87, 213, 270) corresponding 4,8,12-trimethyltridecanoic acid (hereafter TMTD) fragmentation, the second group of ions (*m/z* 74, 88, 101, 312) corresponding to pristanic acid, the third group of ions (*m/z* 74, 101, 171, 326) corresponding to phytanic acid, and the fourth group of ions (*m/z* 74, 105, 262, 290, 318, 346) corresponding to *ω*-(o-alkylphenyl) alkanoic acids (hereafter APAAs) of carbon length C_16_ to C_22_ were monitored, respectively. This permitted the ratio of the two natural phytanic acid diastereomers, 3S,7R,11R,15-phytanic acid (*SRR*) and 3R,7R,11R,15-phytanic acid (*RRR*) to be determined, which can discriminate between aquatic and ruminant organisms (Lucquin et al., 2016). Compounds were identified using Agilent MSD ChemStation and Mass Hunter for Quantitative Analysis (for GC-MS). TLEs were analysed by a HT-DB1 GC-MS-FID, 100% dimethylpolysiloxane (15 m × 0.32 mm × 0.1 μm) (J&W Scientific, Folsom, CA, USA) column. The injector was maintained at 350 °C. The oven temperature was set at 50 °C for 2 min, and then raised by 10 °C/min to 350 °C, where it was held for 15 min. The column flow was split 9:1 (MSD: Flame ionization detector), with the MSD conditions described above.

**Contamination identified during the molecular analyses**

Although contamination in the form of plasticisers (Daktariškė 5, *n* = 1; Gribaša 4, *n* = 2; Karaviškės 6, *n* = 4; Dubičiai 2, *n* = 2; Neravai, *n* = 1; Nida, *n* = 2) and cyclic octaatomic sulfur (Daktariškė 5, *n* = 5; Šventoji 4, *n* = 1) was identified, it has not had an effect on interpretation. Prior to analysis by GC-C-IRMS, the 18 samples containing sulphur were filtered through a pipette with spongy copper and glass wool, solvent rinsed (x 3) and evaporated under a gentle stream of N_2_ at 37 °C. Then, the samples were re-dissolved in 100 μl of hexane and analysed by GC-C-IRMS.

**Gas Chromatography-combustion-Isotope Ratio Mass Spectrometry**

Analysis by GC-C-IRMS was undertaken on the carbon stable isotope ratios of the two fatty acid methyl esters, methyl palmitate (C_16:0_) and methyl stearate (C_18:0_). Prior to analysis, both samples were diluted with hexane. The analysis was performed on a Delta V Advantage Isotope Ratio Mass Spectrometer (Thermo Fisher Scientific, Bremen, Germany) linked to a Trace 1310 Gas Chromatograph (Thermo Fisher) with a ConFlo IV interface (CuO combustion reactor held at 850 °C). 1 μL of each sample was injected into a DB-5 fused-silica column (60 m x 0.25 mm id x 0.25 μm film thickness). The temperature was set for 0.5 min at 50 °C, which increased by 25 °C min^−1^ to 175 °C, 8 °C min^−1^ to 325 °C and held for 20 min. The carrier gas was ultra high purity grade helium at a flow rate of 2 ml min^−1^. The eluted products were combusted to CO_2_ and ionised in the source of the mass spectrometer by electron ionisation. The ion intensities of *m/z* 44, 45, and 46 were monitored in order to automatically compute the ^13^C/^12^C ratio of each peak in the acidified methanol extracts. Computations were performed with Isodat 3.0 Gas Isotope Ratio MS Software (version 3.0; Thermo Fisher), which were based on comparisons with a standard reference gas (CO_2_) of known isotopic composition that was repeatedly measured. The results from the analysis are reported in parts per mille (‰) relative to an international standard (V-PDB).

The accuracy and precision of the instrument was determined on *n*-alkanoic acid ester standards of known isotopic composition (Indiana standard F8-3). The mean ± S.D. values of these were -29.90 ± 0.20‰ and -23.19 ± 0.12‰ for the methyl ester of C_16:0_ (reported mean value vs. VPDB -29.90 ± 0.03‰) and C_18:0_ (reported mean value vs. VPDB -23.24 ± 0.01‰) respectively. Each sample was measured in replicate (mean of S.D. 0.04‰ for C_16:0_ and 0.03‰ for C_18:0_). Values were also corrected subsequent to analysis to account for the methylation of the carboxyl group that occurs during acid extraction. Corrections were based on comparisons with a standard mixture of C_16:0_ and C_18:0_ fatty acids of known isotopic composition processed in each batch under identical conditions.

**References for Supplementary Text and Tables S1 to S7**

Antanaitis I, Ogrinc N (2000) Chemical analysis of bone: stable isotope evidence of the diet of Neolithic and Bronze Age people in Lithuania. Istorija 45:3-12.

Antanaitis-Jacobs I, Richards M, Daugnora L, Jankauskas R, Ogrinc N (2009) Diet in early Lithuanian prehistory and the new stable isotope evidence. Archaeologia Baltica 12:12-30.

Butrimas A (1992) Daktariškės 5 neolito gyvenvietės tyrinėjimai. Archeologiniai tyrinėjimai Lietuvoje 1990 ir 1991 metais: 8-11.

Cramp LJE, Evershed RP, Lavento M, Halinen P, Mannermaa K, Oinonen M, Kettunen J, Perola M, Onkamo P, Heyd V (2014) Neolithic dairy farming at the extreme of agriculture in northern Europe. Proc R Soc Lond 281:20140819. DOI: 10.1098/rspb.2014.0819.

Eriksson G, Howcroft R (2014) Stable carbon and nitrogen isotope analysis of skeletal remains of humans and pigs. In: Przybyła M, Szczepanek A, Włodarczak P (eds) Koszyce, stanowisko 3. Przemoc i rytuał u schyłku neolitu. Wydawnictwo i Pracownia Archeologiczna PROFIL-ARCHEO Magdalena Dzięgielewska, Kraków, pp 109-117.

Eriksson G, Lõugas L, Zagorska I (2003) Stone age hunter-fisher-gatherers at Zvejnieki, northern Latvia: stable isotope and archaeozoological data. Before Farming 1:1-25.

Eriksson G, Linderholm A, Fornander E, Kanstrup M, Schoultz P, Olofsson H, Lidén K (2008) Same island, different diet: Cultural evolution of food practice on Öland, Sweden, from the Mesolithic to the Roman period. J. Anthropol. Archaeol 27(4):520-543. DOI: 10.1016/j.jaa.2008.08.004.

Fornander E (2013) Dietary diversity and moderate mobility - isotope evidence from Scanian Battle Axe Culture burials. Journal of Nordic Archaeological Science 18:13-29.

Grinevičiūtė G (2002) Gribašos 4-oji akmens amžiaus gyvenvietė. Archaeologia Lituana 3:5-33.

Heron C, Craig OE, Lucquin AJA, Steele VJ, Thompson A, Piličiauskas G (2015) Cooking fish and drinking milk? Patterns in pottery use in the southeastern Baltic, 3300-2400 cal BC. J. Archaeol. Sci. 63:33-43. DOI: 10.1016/j.jas.2015.08.002.

Kuncienė O (1978) Naravų (Trakų raj.) pilkapyno tyrinėjimai 1976 ir 1977 metais. Archeologiniai tyrinėjimai Lietuvoje 1976 ir 1977 metais: 126-132.

Laneman M, Lang V (2013) New radiocarbon dates for two stone-cist graves at Muuski, northern Estonia. Estonian Journal of Archaeology 17(2):89-122. DOI: 10.3176/arch.2013.2.01.

Lucquin A, Colonese AC, Farrell TFG, Craig OE (2016) Utilising phytanic acid diastereomers for the characterisation of archaeological lipid residues in pottery samples. Tetrahedron Lett. 57(6):703-707. DOI: 10.1016/j.tetlet.2016.01.011.

Piezonka H, Kostyleva E, Zhilin MG, Dobrovolskaya M, Terberger T (2013) Flesh or fish? First results of archaeometric research of prehistoric burials from Sakhtysh IIa, Upper Volga region, Russia. Documenta Praehistorica XL:57-73. DOI: 10.4312/dp.40.6.

Piličiauskas G (2004) Akmens ir bronzos amžiaus stovyklos Karaviškėse (Karaviškių 6-oji gyvenvietė, plotai II ir IV). Lietuvos Archeologija 25:157-186.

Piličiauskas G (2016) Lietuvos pajūris subneolite ir neolite. Žemės ūkio pradžia. Lietuvos Archeologija 42: 25-103.

Piličiauskas G (2018) Virvelinės keramikos kultūra Lietuvoje 2800–2400 cal BC. Lietuvos istorijos institutas, Vilnius.

Piličiauskas G, Heron C (2015) Aquatic Radiocarbon Reservoir Offsets in the Southeastern Baltic. Radiocarbon 57(4): 539-556. DOI: 10.2458/azu_rc.57.18447.

Piličiauskas G, Gaižauskas L, Kalinauskas A, Peseckas K, Rutavičius J, Piličiauskienė G, (2017a) Nidos akmens amžiaus gyvenvietė. Archeologiniai tyrinėjimai Lietuvoje 2016 metais: 48-52.

Piličiauskas G, Piličiauskienė G, Jankauskas R, Dupras T (2017b) Reconstructing Subneolithic and Neolithic diets of the inhabitants of the SE Baltic coast (3100−2500 cal BC) using stable isotope analysis. Archaeolog. Anthrop. Sci. 9(7):1421-1437. DOI: 10.1007/s12520-017-0463-z.

Piličiauskas G, Jankauskas R, Piličiauskienė G, Craig OE, Charlton S, Dupras T (2017c) The transition from foraging to farming (7000–500 cal BC) in the SE Baltic: A re-evaluation of chronological and palaeodietary evidence from human remains. J. Archaeol. Sci. Rep. 14:530-542. DOI: 10.1016/j.jasrep.2017.06.004.

Piličiauskas G, Asheichyk V, Osipowicz G, Skipitytė R, Varul L, Kozakaitė J, Kryvaltsevich M, Vaitovich A, Lakiza V, Šapolaitė J, Ežerinskis Ž, Pamazanau M, Lucquin A, Craig OE, Robson HK (2018a) The Corded Ware culture in the Eastern Baltic: New evidence on chronology, diet, beaker, bone and flint tool function. J. Archaeol. Sci. Rep. 21:538-552. DOI: 10.1016/j.jasrep.2018.08.023.

Piličiauskas G, Skipitytė R, Heron C (2018b) Mityba Lietuvoje 4500–1500 cal BC maisto liekanų keramikoje izotopinių tyrimų duomenimis. Lietuvos archeologija 44:9-41.

Pospieszny K, Sobkowiak-Tabaka I, Price TD, Frei KM, Hildebrandt-Radke I, Kowalewska-Marszałek H, Krenz-Niedbała M, Osypińska M, Stróżyk M, Winiarska-Kabacińska M (2015) Remains of a late Neolithic barrow at Kruszyn. A glimpse of ritual and everyday life in early Corded Ware societies of the Polish Lowland. Praehistorische Zeitschrift 90(1-2):185-213. DOI: 10.1515/pz-2015-0009.

Reitsema LJ (2012) Stable Carbon and Nitrogen Isotope Analysis of Human Diet Change in Prehistoric and Historic Poland. Dissertation, Ohio State University.

Rimantienė R (1989) Nida. Senųjų baltų gyvenvietė. Mokslas, Vilnius.

Rimantienė R (1999) Pelesos paežerių akmens amžiaus stovyklos ir gyvenvietės. Lietuvos archeologija 16:77-105.

Rimantienė R (2005) Die Steinzeitfischer an der Ostseelagune in Litauen. Litauisches Nationalmuseum, Vilnius.

Robson HK, Andersen SH, Clarke L, Craig OE, Gron KJ, Jones AKG, Karsten P, Milner N, Price TD, Ritchie K, Zabilska-Kunek M, Heron C (2016) Carbon and nitrogen stable isotope values in freshwater, brackish and marine fish bone collagen from Mesolithic and Neolithic sites in central and northern Europe. Environmental Archaeology 21(2):105-118. DOI: 10.1179/1749631415Y.0000000014.

Sjögren K-G, Price TD, Kristiansen K (2016) Diet and Mobility in the Corded Ware of Central Europe. PLoS ONE 11(5):e0155083. DOI:10.1371/journal.pone.0155083.

Tõrv M, Meadows J (2015) Radiocarbon dates and stable isotope data from the Early Bronze Age burials in Riigiküla I and Kivisaare settlement sites, Estonia. Radiocarbon 57(4):645-656. DOI: 10.2458/azu_rc.57.18459.

Vengalis R, Juškaitis V, Pilkauskas M, Kozakaitė J (2016) Kvietinių senovės gyvenvietė ir pilkapynas. Archeologiniai tyrinėjimai Lietuvoje 2015 metais: 74-86.
